# Effects of Long‐Term Exposure to the Earned Income Tax Credit on Work Disability in Later Life

**DOI:** 10.1002/hec.70068

**Published:** 2025-12-05

**Authors:** Katie Jajtner, Keisha T. Solomon, Yang Wang

**Affiliations:** ^1^ Jajtner, Retirement and Disability Research Center University of Wisconsin‐Madison Madison Wisconsin USA; ^2^ Howard University Washington District of Columbia USA; ^3^ La Follette School of Public Affairs University of Wisconsin‐Madison Madison Wisconsin USA

**Keywords:** earned income tax credit, social policy, work disability

## Abstract

This study investigates the impact of the Earned Income Tax Credit (EITC) on work disability and Social Security Disability Insurance (DI) claims among Americans. Utilizing the Panel Study of Income Dynamics, we examine the effects of EITC exposure from birth to mid‐adulthood on work disability risk before retirement. Our analysis reveals that EITC exposure during adulthood significantly reduces the likelihood of work disability, potentially influencing DI trends. Specifically, a $10,000 increase in cumulative EITC exposure is associated with about a 1.25 percentage‐point lower probability of any work limitation at ages 50–61 (a 0.94 percentage‐point reduction in the likelihood of chronic/severe limitations) and a 0.84 percentage‐point reduction in DI receipt, highlighting the EITC's potential role in reducing DI dependency and its broader implications for public policy and social welfare.

## 1 Introduction

Work disability is a condition that constrains the type or amount of work an individual can perform. For eligible Americans who cannot work due to a work‐limiting disability, Social Security Disability Insurance (DI)—a social insurance program—offers some income replacement (Social Security Administration, n.d.). Over 2 million workers apply for DI annually (Social Security Administration 2019). Approximately 7.8 million American workers with disabilities received DI in 2021 (Social Security Administration 2022, Table 19), which totaled $10.6 billion in benefits (Social Security Administration 2022). Understanding the pathways and contexts that improve individual health and well‐being is crucial to mitigating the risk that individuals will need DI. Previous literature demonstrates that health and disability vary across U.S. states (Courtney‐Long et al. 2015). Moreover, social policies like the Earned Income Tax Credit (EITC) also differ across U.S. states and are known to influence individual health and labor force attachment (e.g., Bastian and Jones 2021; Bastian and Michelmore 2018; Braga et al. 2020; Lenhart 2019). In this paper, we explore whether the EITC could affect the probability of reporting work disability among adults aged 50–61 years. Understanding the relationship between the EITC and the likelihood of reporting work disability among middle‐aged adults is important because the risks of developing work disability increase with age, with nearly 80% of DI beneficiaries being older than 49 years (Social Security Administration 2022, Chart 4). We hypothesize that exposure to the EITC throughout an individual's life course could improve health and labor market attachment sufficiently to decrease the probability of work disability later in life.

Existing literature partially recognizes the potential impact public policy may have on work disability. For example, Hoynes et al. (2016) and Goodman‐Bacon (2021) find that exposure to Food Stamps, which was renamed the Supplemental Nutrition Assistance Program in 2008, and Medicaid, respectively, could reduce work disability. However, these studies classify work disability in a limited binary fashion and yield effects that are often imprecise. While the limited existing evidence supports our hypothesis that public policy may reduce the incidence of work disability, further research is required.

We use data from the Panel Study of Income Dynamics (PSID) to examine the effect of the EITC throughout an individual's life course, that is, from birth to mid‐adulthood, on individuals experiencing work disability in the years leading up to standard retirement ages. Using a generalized difference‐in‐differences method, we find that exposure to the EITC during adulthood can substantially and statistically significantly decrease the probability of work disability in the decade before standard retirement ages, and this effect is heterogeneous across various subgroups. To contextualize our findings, we also provide back‐of‐the‐envelope calculations to quantify the economic impacts of increased EITC exposure on DI awards. Our calculations indicate that savings accruing from averted DI awards due to the EITC represent approximately 10 percent of EITC expenditures. This finding enhances the short‐term self‐financing rate reported by Bastian and Jones (2021) of 83%, and lends empirical support to their hypothesis that if the long‐run impacts of the EITC are accounted for, the program could pay for itself.

While existing studies have begun to show the multifaceted impacts of the EITC on health outcomes and labor force participation (see Section 2), a critical gap remains in our understanding of how these effects translate into the domain of work disability. Previous research has predominantly focused on the direct health benefits and employment incentives provided by the EITC (Averett and Wang 2013; Bastian and Jones 2021; Eissa and Hoynes 2006; Evans and Garthwaite 2014; Gangopadhyaya et al. 2020; Lenhart 2019; Meyer 2010; Muennig et al. 2016), underscoring its potential as a tool to lift low‐income families out of poverty and to improve their health and their access to healthcare. However, these studies have not fully explored the relationship between the EITC and subsequent risk of work disability — a condition that significantly impacts individuals' ability to remain in the workforce and poses substantial implications for public health and social insurance systems.

Our study seeks to bridge this gap by carefully analyzing the extent to which long‐term exposure to the EITC can mitigate the likelihood of reporting work disability among adults close to retirement age. In doing so, we respond to a vital yet unanswered question: can a tax policy designed to incentivize low‐ and medium‐income individuals to work and to pull them out of poverty contribute to reducing the incidence of work disability, thereby sustaining workforce participation and lessening the burden on DI? Using a rigorous method and many robustness checks, we find the EITC not only reduces the likelihood of self‐reported work disability, but may also serve as a preventative measure to avert future DI claims. This dual examination of the EITC's implications for health and labor market attachment contributes to our understanding of the intersections between public policy, health economics, and labor force dynamics.

Furthermore, this study makes several unique contributions to the existing literature on social policies and work disability. First, unlike prior research that often treats work disability as a binary outcome, we employ a more nuanced approach by examining four distinct constructs of work disability, capturing its varying duration and severity. Second, we extend the scope of analysis to cover policy exposure throughout an individual's life course—from birth to mid‐adulthood—providing a more comprehensive understanding of the long‐term effects of policies like the EITC. Finally, our work is among the first to propose a holistic approach to understanding how social policies may serve dual roles as both safety nets and preventative measures, thereby offering valuable insights for both researchers and policymakers.

Our study also has several important policy and research implications. First, our findings underscore the need to evaluate how public policies impact health and work disability over the life course, not only during childhood. Second, our aim to examine the long‐term impact of a social policy that was first rolled out in the 1970s highlights the importance of adopting a long‐term perspective in the planning and evaluation of public policies. Third, our research suggests that social programs could function as both safety nets and preventative measures to maintain the health and labor force participation of the population. Fourth, it is crucial for researchers to recognize the multi‐faceted nature of work disability to provide policymakers with a more informative lens for understanding how policy can shape DI applications and awards. Finally, our study's goal to provide a more holistic understanding of potential policy spillovers underscores the need for comprehensive data collection and analysis that incorporates multiple factors across various life stages. This could influence data collection policies and strategies to better capture this information for research and policy‐making purposes.

The rest of the paper is organized as follows. Section 2 provides background information on DI, work disability, and the EITC. Section 3 outlines the theoretical framework underpinning our empirical work. In Section 4, we describe our data and empirical method. Section 5 presents our results, and we discuss and conclude in Section 6.

## 2 Background

### 2.1 Social Security Disability Insurance

DI provides crucial income support to individuals who are unable to work due to significant, medically determinable physical or mental impairments. Eligibility for DI is stringent, with applicants required to meet both work history and medical condition criteria. Specifically, individuals must have contributed to Social Security through work for 40 quarters, that is, 10 years, and five of the last 10 years before their disability. The medical impairment must be severe enough to last at least 12 months or result in death, and it must prevent the applicant from performing any substantial gainful activity (Social Security Administration 2024). DI covers a broad spectrum of physical and mental impairments, with musculoskeletal conditions and severe mental disorders being prominent among beneficiaries (Social Security Administration 2022, Chart 6). Applicants undergo a rigorous evaluation process, starting with a technical assessment to verify work history eligibility, followed by a medical evaluation conducted by state disability determination services (Social Security Administration (SSA) 2018).

DI benefits are calculated from a worker's average indexed monthly earnings using Social Security's progressive primary insurance amount formula; disabled workers are entitled to 100% of that amount. In practice, this yields benefits that rise with prior earnings but replace a limited share of pre‐disability wages. The average monthly disabled‐worker benefit was $1537 in December 2023^1^ and $1581 in December 2024.^2^ Benefits typically begin after a 5‐month waiting period,^3^ and Medicare coverage generally begins 24 months after benefits start.^4^ Beneficiaries must also remain below substantial gainful activity (SGA) thresholds, which SSA sets annually. For 2024, the SGA amounts are $1550/month (non‐blind) and $2590/month (blind), rising to $1620 and $2700 in 2025.^5^ These rules mean DI provides crucial income insurance when earnings capacity falls below SGA, but for individuals who can sustain work above SGA, especially in less physically demanding roles, continued employment may yield higher monthly resources than DI.

Since its inception, the size of the DI program, that is, the number of DI beneficiaries, has changed over time. These changes may be driven by amendments to the program, increased public awareness of the program, economic conditions, and demographic changes. For example, the reductions in the number of DI beneficiaries from the mid‐1970s to the early 1980s may have resulted from legislation by Congress to reduce DI benefits (Social Security Administration, n.d.). Following the reduction of the DI program in the 1980s, there was historical growth in DI beneficiaries, peaking from the 1990s through the early 2010s. This growth may have been driven by demographic factors such as population growth, aging, and increased labor force participation among women, particularly those from the baby‐boom generation. Economic conditions, notably recessions, can also impact DI applications and awards (Maestas et al. 2021) After the significant rise in beneficiaries, DI enrollment has been in decline since 2014, with a notable drop in both applications and awards.

### 2.2 Work Disability

Work disability affects millions of Americans. Although not precisely defined as work disability by the SSA, household surveys often inquire whether respondents have a health condition that limits the type or amount of work they can perform. The data we use allow follow‐up questions to help gauge the duration and severity to better approximate chronic and severe conditions more likely to qualify for DI benefits from SSA. According to the SSA, 25% of 20‐year‐olds will experience a disability before retirement (Social Security Administration, n.d.). One study that uses the same survey data as our study estimates the prevalence of chronic and severe work disability to be 26% by age 60; however, including temporary and short‐term conditions, the estimates of American male household heads experiencing the condition by age 60 skyrocket to just under 70% (Meyer and Mok 2019).

The experience of work disability is linked with several disadvantages. Even prior to the onset of work disability, individuals report a substantial earnings drop (Charles 2003) that persists for years following onset (Jolly 2013; Meyer and Mok 2019). In addition to lower earnings, those with work disability or limitations also experience food insecurity, economic hardships such as the inability to meet essential expenses, higher poverty rates, and adverse aging experiences (Jajtner et al. 2022; Mitra et al. 2020; She and Livermore 2007; Shuey and Willson 2017). Thus, work disability is both common and represents an enormous economic and material well‐being hardship.

The essence of work disability lies at the intersection of health and labor market participation. When a health condition is present that limits or prevents work, work disability can result with unaccommodating environments. Social policies can have meaningful impacts on health, the labor market, and economic outcomes for recipients (Bastian and Michelmore 2018; Boudreaux et al. 2016; Braga et al. 2020; Goodman‐Bacon 2021; Hoynes et al. 2016; Hoynes and Patel 2018; Jones 2020; Miller and Wherry 2019). Thus, we hypothesize (broadly) that social policies could reduce the likelihood of experiencing work disability. Specifically, we consider whether the EITC could mitigate the prevalence of work disability.

### 2.3 Institutional Background on the EITC

The EITC began in 1975 with a modest maximum federal credit of $400 for low‐ and medium‐income households with children ($1924 in 2020 dollars). It has a pyramid design where the credit increases with earnings, reaches a maximum plateau, and then phases out as earnings continue to increase. The initial policy goals included alleviating tax burdens, incentivizing work, and increasing employment. All households with children, regardless of size or location, were eligible for the same federal credit of $500 in 1982 (about $1341 in 2020 dollars).

Initially a federal‐only program, the EITC has since been adopted by many states. As of 2023, about 31 states and the District of Columbia offer their own EITC, typically set as a percentage of the federal credit, ranging from modest (e.g., 3% in some states) to generous (up to 125% in South Carolina). The year 1983 marked the beginning of a rich history of changes and variations in the EITC. Wisconsin pioneered the first state EITC policy in 1983 to complement the federal benefit, adding approximately $390 to the federal credit in 2020 dollars. By 1990, Iowa, Maryland, Rhode Island, and Vermont had joined Wisconsin in offering a state EITC. In 1991, the federal credit began to vary by family size, where families with two or more children received a 3.6% boost to their maximum credit. State credits, mainly a set percentage of the federal credit, mirrored this change and amplified variation by geography and family size across the nation. Just three years later, the maximum federal credit for households with two or more children was about 24% larger than the benefit available to households with a single child, and childless workers became eligible for a small EITC benefit. The 1995–2008 era witnessed relative stability in federal real EITC benefits hovering around $5000 ‐ $6000 for families with two or more children. The bulk of variation in this era is attributable to 15 new states and the District of Columbia establishing their own EITCs, bringing the total number of states with credits to 22 states plus the District of Columbia. The final significant federal policy change occurred in 2009 when the EITC benefit differentiated credits for families with three or more children relative to smaller families. By 2017, the final year of EITC data for the youngest cohort in our main study sample, the real federal benefit in 2020 dollars stood at $538 for individuals without dependents and $6671 for those with three or more children. During this year, 25 states and the District of Columbia offered EITC. Some state EITCs are refundable, meaning families can receive full benefit even if it exceeds their tax liability, while others are non‐refundable and only offset taxes owed.

The EITC is a unique social safety net program, heralded as one of the most effective anti‐poverty programs (Scholz 1994; Simon et al. 2018). It has been argued to have remarkably low net budgetary costs in the long run when considering induced changes in labor supply and reduced public assistance uptake (Bastian and Jones 2021). However, in the short run, the program does require substantial expenditures. There are two main avenues through which the EITC could influence work disability. First, a large body of research demonstrates that the EITC meaningfully increases labor market participation and income (Bastian 2020; Bastian and Jones 2021; Bastian and Michelmore 2018; Dickert et al. 1995; Eissa and Hoynes 2006; Grogger 2003; Hoynes 2009; Meyer 2010). This effect contributes to increased tax revenues and decreased government transfers, offsetting approximately 83% of the budgetary cost in one year (Bastian and Jones 2021). The impact of increased labor market participation due to the EITC on work disability is somewhat ambiguous. On one hand, when labor market participation increases on an extensive margin, individuals newly entering the labor market can become mechanically eligible to experience work disability. At least some of these individuals are likely to experience health conditions that subsequently limit their labor market participation later in life. On the other hand, work itself could offer health benefits, particularly psychological ones (Jahoda 1981; Zechmann and Paul 2019), that could decrease the likelihood of work disability.

Second, research also demonstrates that the EITC can meaningfully alter recipients' health (Averett and Wang 2013; Cowan and Tefft 2012; Evans and Garthwaite 2014; Gangopadhyaya et al. 2020; Lenhart 2019; Muennig et al. 2016). For example, one study suggests that an additional $1000 in EITC benefits can lead to a 2‐ to 3‐percent reduction in the incidence of low birth weight (Hoynes et al. 2015). EITC benefits are reported to improve self‐reported health status (Braga et al. 2020; Lenhart 2019) and decrease the probability of obesity (Braga et al. 2020), and mothers receiving EITC benefits also report fewer days with poor mental health (Evans and Garthwaite 2014). These documented health improvements throughout the life course due to the EITC should reduce the likelihood of work disability by diminishing the prevalence of work‐limiting health conditions. Considering both labor and health effects, we anticipate that the EITC will decrease the likelihood of work disability on net, although this is ultimately an empirical question.

## 3 Theoretical Motivation

The relationship between social safety nets and health outcomes is a subject of considerable scholarly debate, dating back to seminal works like those of Arrow (1963). The prevailing theoretical framework posits that social safety nets, such as the EITC, can function as economic buffers with potential downstream effects on health. This relationship can be conceptualized through the lens of Grossman's Health Production Function Model (Grossman 1972), which offers a comprehensive framework to explore the intricate relationships between social safety nets and work disability. In his seminal work, Grossman posited, first, that individuals derive utility not only from the consumption of goods and leisure but also from health, and second, that individuals are both producers and consumers of health. They allocate time and resources to produce “health capital,” which depreciates over time but can be bolstered by “investments” in the form of not only medical care but also other “goods,” such as income, education, and environmental factors.

### 3.1 EITC, Work Disability, and the Grossman Model

We employ Grossman (1972)'s health capital framework to conceptualize how EITC can influence long‐run health and, in turn, the risk of work disability. In this framework, an individual's health capital (*H*_*t*_) at time *t* is treated as a durable stock that produces utility (as a consumption good) and enhances productivity (as an investment good). Health capital depreciates overtime at an exogenous rate *δ*_*t*_ (e.g., due to aging or wear‐and‐tear), and individuals can invest in their health (*I*_*t*_) by devoting resources (time, medical care, nutrition, etc.) to improve or maintain it. Health is also subject to random shocks *ε*_*t*+1_ (e.g., injuries or illnesses) that can suddenly reduce the stock. The evolution of health can be summarized by a law‐of‐motion:

$$H_{t+1}=\left(1-\delta_{t}\right)\;H_{t}+f\left(I_{t};\;A_{t}\right)-\varepsilon_{t+1}$$

Here *f*(*I*_*t*_; *A*_*t*_) is a health production function capturing the gains from investment (with *A*_*t*_ representing the efficiency of health production, given technology or environment). This equation highlights that the next period's health depends on the remaining health after depreciation (1−*δ*_*t*_)*H*_*t*_, plus the health gains from current investment, the environment, minus any losses from unforeseen health shocks.

Work disability can be interpreted in this context as the outcome of insufficient health capital: an individual becomes work‐disabled if health *H*_*t*_ falls below the threshold required to work, due to some combination of high depreciation, inadequate investment or environment, and/or severe shocks.^6^ In other words, work disability reflects a failure to maintain enough health capital for sustained employment. Importantly, *δ*_*t*_ is treated as an exogenous factor (e.g., increasing with age); in our framework, social policies like the EITC do not directly change the biological depreciation rate but instead influence health through their effects on *I*_*t*_, *A*_*t*_, and the exposure to shocks.

We incorporate the EITC into this health capital model to identify pathways through which it may affect health and disability outcomes. Let *E*_*t*_ denote an individual's EITC at time *t*. The EITC is a substantial income supplement for low‐ and medium‐income working families and an incentive for labor force participation. The presence of *E*_*t*_ effectively alters the individual's budget constraint and time allocation, which can impact health capital accumulation through several mechanisms.

1. Resource Channel: Increased Health Investment. A larger EITC (*E*_*t*_) loosens the budget constraint, enabling individuals to afford more and better‐quality health investments. Additional income from the credit can be spent on medical care, healthier food, safer housing, and other health‐promoting goods. Moreover, by incentivizing work, the EITC can raise earned income (and often provide access to employer‐sponsored health insurance), further increasing the resources available for health maintenance. Together, these effects allow for higher *I*_*t*_ in the health production function, leading to a larger health stock *H*_*t*+1_ and potentially lowering the long‐run probability of work disability.
2. Time Allocation Channel: Changes in Health‐Production Time. The EITC can also alter how individuals allocate their time, which has ambiguous effects on health. On one hand, by making work more financially rewarding, the EITC may induce individuals (especially secondary earners or single parents) to work more hours. Increased labor hours mean less time available for health‐producing activities such as exercise, sufficient sleep, or attending medical appointments, which can reduce effective health investment. On the other hand, the EITC's income support might allow some individuals to reduce excessive work burdens (for instance, working one job instead of two) or to invest in time‐saving services, thereby freeing up some personal time. Additionally, in certain phases of the EITC schedule (e.g., the phase‐out range), the incentive to earn additional income is lower, which could discourage extra work at the margin and potentially increase leisure or rest time.
3. Health Environment Channel: Improved Healthcare Access and Reduced Stress. In addition to the spending power, the EITC can improve the effectiveness of health investments by enhancing the individual's health environment. Greater financial stability and workforce attachment can translate into better access to healthcare (e.g., through employer‐provided insurance or having funds for preventive care) and healthier behaviors and living conditions. For example, a family receiving a larger EITC might afford more nutritious food and safer neighborhood conditions—inputs that increase the productivity of health investment (higher *A*_*t*_ in the health production function). Extra income can also make it easier to avoid or reduce harmful behaviors (such as smoking or opioid use) that degrade health. Furthermore, improved financial security due to the EITC may alleviate chronic stress (Cohen and Janicki‐Deverts 2012; Sareen et al. 2011), which is an important determinant of both mental and physical health outcomes (Lupien et al. 2009; O’Connor et al. 2021). By fostering a more supportive environment for health maintenance, these indirect effects of the EITC help individuals preserve their health capital and may reduce the likelihood of severe health shocks.

This theoretical framework illustrates that the impact of EITC exposure on later‐life work disability is the net result of multiple channels. Some channels, primarily the income‐mediated increases in health investments and improved health environment, push toward better health and lower disability risk, while other channels, such as reduced time for health production, could work in the opposite direction. The overall sign and magnitude of the EITC's effect on work disability is therefore an empirical question.

### 3.2 Research Questions Based on the Theoretical Model

Applying Grossman's health capital model provides a theoretical lens to analyze how EITC can influence work disability. It allows for a deeper understanding of the causal mechanisms at play and offers a foundation upon which to interpret empirical results, enriching both the empirical rigor and policy relevance of the research. Our study, based on this theoretical foundation, aims to provide a better understanding of how EITC policies could contribute to improved health outcomes and reduced work disability rates in later adulthood by answering the following research questions.

1. Does the EITC lower the probability of work disability in later life? If so, the EITC can be viewed as a public health investment tool that works by bolstering individual health capital, thereby reducing future healthcare and social security costs.
2. Is the impact of the EITC on work disability, an important indicator of health capital, heterogeneous across different demographic groups, considering that these groups might have different health production functions? e.g., one could posit that different racial or ethnic groups, as well as different genders, have varying efficiencies in turning EITC exposure into improved health outcomes (i.e., reduced work disability). Individuals might also have different initial endowments of health capital due to various social determinants of health or face different constraints (e.g., budgetary or informational) that affect their ability to invest in health.
3. At which life stages is EITC exposure most effective in preserving health capital, as evidenced by lower rates of work disability in later life? For example, it is possible that the health production function itself varies over the life course and is more “sensitive” to investment (like EITC exposure) at certain critical periods. Health capital depreciation rates might also differ by life stage.

## 4 Data and Methods

### 4.1 Data

The Panel Study of Income Dynamics (PSID) is uniquely suited to answer our research questions. Started in 1968, the PSID is the world's longest‐running longitudinal household survey, with approximately 18,000 individuals from 5000 American families (PSID 2022). Annually, and biennially since 1997, it has continued to collect economic, health, and demographic data on these individuals and their descendants. In addition to this powerful household survey, we also utilize reported EITC benefits from the University of Kentucky Center for Poverty Research (University of Kentucky Center for Poverty Research 2022).

Our sample consists of PSID individuals born between 1933 and 1969: cohorts that have witnessed significant variation in EITC exposure at various points in their life courses. The PSID is an ideal dataset for examining the effects of the EITC on work disability for several reasons. First, the PSID began shortly before the inception of the EITC, allowing us to directly observe characteristics crucial for defining policy exposure. Furthermore, the PSID's impressively long duration of over 50 years enables us to observe instances of work disability during later adulthood, which we define as ages 50–61. Our main analysis uses the PSID core interview waves 1968–2019. We exclude 2021 from the primary sample because it was fielded during the COVID‐19 pandemic with a new web‐based mode and exhibits atypical reporting patterns,^7^ particularly in work disability. In a robustness check, we re‐estimate the main specifications including data from the 2021 wave, and the results are virtually unchanged (see Section 5.4).

For clarity on age‐to‐wave coverage, the 1933 birth cohort is between ages 50–61 in the 1983–1994 PSID waves (annual interviewing through 1997), whereas the 1969 cohort reports work disability only in 2019, at age 50, in our main sample. Earlier cohorts can have up to 12 observations between ages 50–61, while younger cohorts have fewer observations because they are younger and PSID interviews became biennial after 1997. Our analytic sample includes all individuals born 1933–1969 who are observed at least once between ages 50–61. The resulting panel is unbalanced, with younger cohorts observed in fewer waves and at earlier ages within the 50–61 window. To demonstrate that results are not driven by this imbalance, we report a robustness check that excludes the 1959–1969 cohorts, ensuring full observation through age 61 by 2019, and the results are very similar (see Section 5.4).

### 4.2 Outcome Variables

The outcome of interest is whether an individual acquires a work disability during later adulthood. We utilize several PSID survey questions to ascertain the presence and degree of work disability. First, household heads and their spouses/partners began reporting work limitations consistently in 1972 and 1981, respectively, from a two‐to three‐question series. In each survey wave, individuals are asked, “Do you have any physical or nervous condition that limits the kind or amount of work you can do?” Those who answer “no” are categorized as “non‐limited” for that wave. Individuals who answer affirmatively are asked follow‐up question(s) to gauge severity. Prior to 1986, individuals simply indicated if the health condition limited work “a lot,” “somewhat,” or “just a little/not at all.” We categorize individuals with these responses as having severe, moderate, or mild work limitations in each wave, respectively. Beginning in 1986, work‐limited individuals are further asked whether their limitation prevents certain types of work, with affirmative answers categorized as having severe limitations (this approach follows Meyer and Mok 2019). Our first definition of work disability includes all individuals who ever report any work limitation between ages 50 and 61.

Moving beyond this elementary definition of work disability, we leverage the panel structure of PSID to capture both the duration and the severity of work limitations. We hypothesize that individuals with both chronic and severe limitations are most likely to meet the Social Security Administration's criteria for work disability, and to not only apply for, but also receive benefits. Adapting the categorization in Meyer and Mok (2019), we examine a second definition of work disability that only includes both chronic and severe conditions. To meet this definition of work disability, individuals must report work limitations in at least 25% of the waves in which they are observed between ages 50 and 61, with at least 50% of the positive work limitation reports being severe in nature.^8^ Because the threshold is proportional to observed waves, it scales with panel length. For example, with just two observed work disability reports in the 50–61 age range, a single severe work disability report would classify the individual as having a chronic and severe limitation.

In addition to these binary outcomes, we also use a work limitation index (Jajtner 2020) that captures the duration and severity of self‐reported limitations on a scale from zero to one, where one indicates the most chronic and severe limitations.^9^

Many individuals who report work limitations in household surveys never claim DI. Unfortunately, directly observing DI benefits in the PSID is infeasible during the late 1990s and early 2000s. Therefore, we leverage a feature of the DI program where beneficiaries are eligible for Medicare after a 2‐year waiting period. Because Medicare is primarily available to DI beneficiaries and older Americans aged 65 and above, we use early reports of Medicare coverage before age 65 to proxy for DI receipt.

One concern is that younger cohorts are observed earlier within ages 50–61, when work disability prevalence is lower, which may reduce the likelihood that they ever report a limitation. We explicitly acknowledge this age‐gradient issue and show in a robustness check that restricts the sample to cohorts fully observed through age 61 by dropping the 1959–1969 cohorts, so all retained cohorts can be followed through age 61 by 2019. This robustness check yields estimates that are similar in sign and magnitude to the main results (see Section 5.4, Table 3).

Additionally, the PSID does not provide a consistent measure of age at first onset for the work‐limitation outcome. The standard question on work‐limiting health conditions is fielded each wave, but a follow‐up question regarding onset was only asked in early waves and subsequently discontinued. As a result, we cannot uniformly recover the onset age. Some respondents also report being limited from their first PSID interview, implying onset prior to panel entry. To ensure correct temporal ordering in our analysis, we (i) define EITC exposure cumulatively through age 49 and examine work‐limitation outcomes at ages 50–61, and (ii) conduct robustness checks that drop any individual reporting a work limitation (or a severe limitation) before age 50 (see Table 3). These design choices ensure, by construction, that policy exposure is measured prior to disability onset in the restricted samples.

Our main outcome variables measure self‐reported work‐relevant functional capacity. The PSID question on the presence of a work‐limiting health condition directly targets the intersection of health and employment, which aligns with SSA's disability definition. Meyer and Mok’s (2019) persistence/severity approach, which we follow, requires repeated reports and high severity so that transient ailments or borderline cases are not misclassified as lasting work disability. This yields a conceptually tight proxy for clinically meaningful and economically consequential limitations in the ability to work, consistent with our focus on later‐life labor capacity. Because self‐reports are subjective in nature, we additionally report effects of the EITC on likely DI claims and supplement these work‐disability measures with additional objective outcomes in Activities of Daily Living (ADLs) and the presence of activity‐limiting physician‐diagnosed health conditions (see Section 5.4).

### 4.3 Measure of EITC Exposure

We measure respondents' exposure to the EITC using statutory policy parameters rather than realized/received EITC benefits. For each person‐year from birth through age 49, we compute the maximum federal EITC (plateau amount) implied by the federal schedule in that calendar year and the household's number of qualifying children, and then add the state supplement.^10^ This yields an annual policy‐generosity amount that varies by year, state of residence, and household child count, but not by actual earnings or tax liability. We then sum annual amounts over ages 0–49 and express the total in 2020 USD (CPI‐U). This approach captures exogenous variation in the EITC policy environment and avoids endogeneity from health‐driven earnings or take‐up and is a common strategy in the EITC literature (e.g., Hoynes et al. (2015) use the maximum EITC credit by year and family size as their policy measure).

EITC exposure is computed at the family‐unit level and assigned to all members of the PSID family unit (head, spouse/partner, and related household members). The qualifying‐child count follows the contemporaneous IRS age/status rules embedded in the policy schedules we use.^11^ Any time an individual enters or leaves a household, exposure reflects the current family unit's qualifying‐child count that the head and spouse would likely be able to claim for tax purposes. This assignment treats the EITC as a household resource shock shared within the family unit each year.

### 4.4 Sources of Identification

Identification of the causal effects of exposure to the EITC from birth to mid‐adulthood on work disability before retirement comes from a large number of variations in EITC benefits due to plausibly exogenous EITC policy changes in our sample period. These variations arise from two sources. First, the EITC was only a federal program when it started in 1975. By March 2024, however, 31 states and the District of Columbia have implemented their own EITC (between‐state variation). State EITC rates are typically a percentage of the federal rate and vary dramatically from 3% (Montana) to 125% (South Carolina) of the federal credit (NCSL 2024), and states have been changing their EITC rates frequently (between‐state variation). Second, the federal government has expanded the EITC program via increased benefit generosity and differentiating benefits by family composition several times since its inception. Because state EITC rates are typically a varying percentage of the federal rate, these federal expansions created differential increases in EITC benefits for families of different sizes in different states (within‐state variation). Exploring these variations leverages the full set of policy changes that occurred during our sample period (over 4 decades), allowing for a more precise estimation of the impact of exposure to EITC on work disability.

In our context, the largest source of identifying variation comes from federal EITC schedule changes that differ by the number of qualifying children (i.e., 0, 1, 2, and 3 or more). State supplements provide additional, but smaller, variation via state‐specific percent‐of‐federal multipliers largely because the birth cohorts in our sample are older.

### 4.5 Modeling

The effect of EITC exposure throughout an individual's life course on later‐life work disability is analyzed using a generalized difference‐in‐differences model as in Equation 1. Thus, we identify the effects of EITC exposure on work disability by exploiting variations in EITC benefits across states and over time. This method is particularly well‐suited for our study for several reasons. First, it allows us to explore all the temporal and geographical variations in EITC policies in our sample period (detailed above in Section 4.4), essentially mimicking a natural experiment. This approach is crucial for estimating causality in the absence of randomized controlled trials, which is often impractical in policy research. Second, this method controls for state‐level time‐invariant unobservable characteristics that could confound the relationship between EITC exposure and work disability outcomes, such as innate health predispositions or long‐term economic conditions in a given state. Lastly, the generalized difference‐in‐differences framework is sufficiently flexible to accommodate multiple treatment groups and time periods, making it ideal for a study that examines policy effects across different life stages.

In Equation (1) below, the coefficient of interest is $\beta_{1}$: the effect of EITC exposure for individual *i* before late adulthood (i.e., ages 0–49) on work disability in late adulthood (i.e., ages 50–61). We first examine the effect of life course exposure to the EITC before 50 years old (i.e., ages 0–49). Second, we examine the effect of EITC exposure in childhood and adulthood (i.e., ages 0–18 and ages 19–49) separately to determine if exposure during either of these distinct life stages might be more influential. In the latter case, $\beta_{1}$ is a vector of coefficients representing those two phases of the life course before late adulthood. Note that because our EITC exposure is a continuous measure in thousands of 2020 USD, the coefficient *β* is the marginal effect of a $1000 increase in EITC exposure, conditional on all the controls and fixed effects in Equation (1).

(1) $${Work\;Disability}_{i,50-61}=\beta_{0}+\beta_{1}{\text{EITC}}_{i,0-49}+\sum_{j}\beta_{j}X_{ij}+\sum_{n}\beta_{n}V_{in}+\sum_{s}\beta_{s}Z_{is}+\delta +\gamma +\varepsilon_{i}$$

Following the existing literature (Bastian and Michelmore 2018; Boudreaux et al. 2016; Goodman‐Bacon 2021; Hoynes et al. 2016), we additionally control for the following individual factors over ages 50 to 61, which we denote as period “*j*” of individual *i*'s life course, ($\sum_{j}\beta_{j}X_{ij}$): age, age‐squared, race/ethnicity, educational attainment, marital status, poverty, and fixed‐effect indicators for the maximum number of EITC‐eligible children in adulthood (four mutually exclusive categories: 0, 1, 2, 3+).^12^ Individual characteristics before late adulthood, which we denote as period “*n*”, ($\sum_{n}\beta_{n}V_{in}$) include the portion of time spent in poverty (ages 0–49) and the portion of time an individual is married during adulthood (ages 30–49). Finally, to buttress results against potential contamination by unobserved state characteristics in each state, *s,* we control for several state characteristics observed before age 50 ($\sum_{s}\beta_{s}Z_{is}$). Specifically, we use data from Goodman‐Bacon (2021) on state income per capita and the number of hospital beds per capita, data on the state‐level unemployment rate from the University of Kentucky Poverty Research Center, and data on minimum wage from the Federal Reserve Economic Data. Following the specification in Hoynes et al. (2016), birth cohort fixed effects (δ) and birth state fixed effects (γ) are also included. Binary measures of work disability are analyzed using a linear probability model, while continuous measures of work disability are estimated using ordinary least squares regression.

We cluster standard errors by the first observed state of residence in all main specifications. Clustering by state is appropriate because the identifying variation comes from state‐by‐year policy variation, and we allow for arbitrary within‐state correlation over time in unobservables. In robustness checks, we show that alternative inference choices leave point estimates essentially unchanged.

### 4.6 Endogeneity Concerns and Solutions

First, an individual's decision to participate in the labor market is at least partially tied to their health. For example, Hokayem and Ziliak (2014) find that men reporting “very good” or “excellent” health on average work 94 h more per year and earn $6.22 more per hour than those who report “poor,” “fair,” or “good” health. As work disability is defined as a health condition that limits work ability, healthier individuals could be more likely to receive EITC benefits (due to their ability to work). To robustly address this potential selection bias—a concern inherent in studies examining the effects of social policies on health and other outcomes—we focus on policy ‘exposure’ rather than actual ‘participation’ in the EITC. Specifically, following the literature (Bastian and Jones 2021; Bastian and Lochner 2020; Bastian and Michelmore 2018; Hoynes et al. 2015) and as described in Section 4.3, we measure EITC exposure using the maximum potential benefits an EITC‐eligible family could receive. Changes in maximum EITC benefits reflect plausibly exogenous policy variations at federal and state levels that are independent of family income, EITC eligibility, or actual EITC benefits received, which are potentially endogenous to labor market decisions. Using maximum EITC benefits also allows us to take advantage of all the policy changes in EITC and to study whether this program as a whole—apart from individual federal expansions or state supplementations—affects work disability for adults near retirement. Note that even though few households actually receive the maximum EITC in any given year, the literature has found that the maximum EITC benefits are highly correlated with other parameters of the EITC and can capture those policy changes in the EITC over time very well (Bastian and Lochner 2020).

By using the maximum EITC benefits, we mitigate the risk that healthier individuals who are more likely to work (and thus receive EITC benefits) could artificially skew the results. That is, measuring exposure rather than actual EITC benefits received allows us to establish a more causal relationship between EITC policies and work disability, independent of an individual's propensity to participate in the program, thus enhancing the internal validity of our findings and reinforcing the case for policy implications.

Second, Bastian and Jones (2021) provide some evidence that state‐level EITC policies could be endogenous with economic conditions and other state policies. Additionally, most states with an EITC policy offer a flat percentage state policy based on the federal benefit, although a few states deviate from this practice, adding some additional variation to the total EITC exposure measure we employ. To address these potential issues, we alternatively estimate our models using only federal EITC exposure, and robust results are reported in Section 5.4.

Third, there is always the concern that individuals eligible for the EITC and other safety‐net measures may migrate to states with more generous policies, thus calling into question the exogenous nature of the exposure (Moffitt 1992). Therefore, in one robustness check, we exclude those who moved across state lines before age 50, and the results of this check, which are qualitatively similar to the main results, are also reported in Section 5.4.

Finally, the PSID's sampling design might lead to selection bias if the selection of participants into our study is related to our outcome of interest (work disability) in a way that is not fully accounted for in the model (e.g., PSID sampling irregularities in the Survey of Economic Opportunity (SEO), Brown 1996). Excluding the SEO from our sample, or unweighting our sample, yields robust results (see Section 5.4).

### 4.7 Sample Descriptive Statistics

Our analysis sample includes the 1933–1969 birth cohorts, with the 1969 birth cohort turning 50 in 2019, the final year of pre‐COVID‐19 pandemic data for self‐reports of work disability.^13^ The 1933 birth cohort is chosen as the earliest cohort because these individuals turned 50 in 1983, the first year of state variation in EITC exposure. Table 1 reports the descriptive statistics for these samples. Column 1 is the sample average for the overall sample, column 2 is the sample average for individuals never reporting a work disability in late adulthood (ages 50–61), and column 3 is the sample average for individuals ever reporting a work disability during that time. Columns 4 and 5 split the work disability sample into those with chronic and severe limitations (column 5) and those with non‐chronic or non‐severe limitations (column 4). The final two columns split the full sample by whether the individual is a likely DI recipient (i.e., has Medicare before age 65). The sample for these last two columns is smaller because Medicare receipt is not reported until 1999. Notably, women and individuals with lower educational attainment are (slightly) overrepresented in the work‐limitation sample. Individuals experiencing work limitations between ages 50 and 61 also have significantly lower household incomes and poorer health, indicated by lower self‐reported health status and a greater portion of later adulthood spent with a limitation in Activities of Daily Living (ADL).

**TABLE 1:** Descriptive statistics.

	(1)	(2)	(3)	(4)	(5)	(6)	(7)
	Sample	Never work limited	Ever work limited	Non‐chronic or non‐severe work limit	Chronic and severe work limit	No DI	DI
% females	0.537	0.512	0.581***	0.589***	0.563+	0.524	0.566+
	(0.008)	(0.011)	(0.013)	(0.016)	(0.024)	(0.010)	(0.022)
% nH white	0.737	0.742	0.729	0.765	0.644***	0.763	0.603***
	(0.007)	(0.009)	(0.012)	(0.013)	(0.023)	(0.008)	(0.021)
% < HS	0.092	0.069	0.134***	0.100**	0.211***	0.062	0.164***
	(0.004)	(0.005)	(0.009)	(0.009)	(0.019)	(0.004)	(0.016)
% HS/GED	0.307	0.294	0.331*	0.322	0.352*	0.289	0.357**
	(0.008)	(0.010)	(0.013)	(0.015)	(0.024)	(0.009)	(0.021)
% some college	0.252	0.246	0.263	0.265	0.260	0.249	0.298*
	(0.007)	(0.009)	(0.012)	(0.015)	(0.022)	(0.008)	(0.021)
% college	0.348	0.391	0.272***	0.313***	0.177***	0.400	0.181***
	(0.008)	(0.010)	(0.012)	(0.015)	(0.019)	(0.010)	(0.018)
Average income (ages 30–49)	$101,245	$110,661	$84,492***	$93,004***	$64,658***	$109,570	$68,875***
	(1360)	(1819)	(1847)	(2283)	(2816)	(1674)	(2136)
Average overall health (ages 50–61)	77.718	84.613	65.451***	71.793***	50.674***	81.235	61.377***
	(0.289)	(0.219)	(0.569)	(0.567)	(1.028)	(0.270)	(1.007)
% time limiting health condition (ages 50–61)	0.209	0.100	0.403***	0.360***	0.503***	0.179	0.480***
	(0.005)	(0.005)	(0.010)	(0.012)	(0.019)	(0.006)	(0.017)
% with any ADL limitation (ages 50–61)	0.224	0.053	0.528***	0.416***	0.788***	0.157	0.623***
	(0.007)	(0.005)	(0.014)	(0.016)	(0.019)	(0.007)	(0.022)
% time ADL limitation (ages 50–61)	0.085	0.015	0.210***	0.138***	0.377***	0.053	0.289***
	(0.003)	(0.002)	(0.008)	(0.007)	(0.016)	(0.003)	(0.014)
EITC exposure (ages 0–18)	$4539	$5403	$3002***	$2814***	$3442***	$4770	$3965**
	(114)	(151)	(156)	(183)	(297)	(127)	(279)
EITC exposure (ages 19–49)	$49,571	$54,132	$41,457***	$42,348***	$39,380***	$52,627	$47,017**
	(639)	(833)	(936)	(1136)	(1635)	(723)	(1555)
EITC exposure (ages 0–49)	$54,111	$59,535	$44,459***	$45,162***	$42,822***	$57,397	$50,982***
	(710)	(928)	(1021)	(1240)	(1785)	(799)	(1703)
Maximum number of dependent children in adulthood	2.035	2.025	2.053	2.057	2.042	2.024	2.009
	(0.016)	(0.020)	(0.028)	(0.033)	(0.054)	(0.019)	(0.047)
% federal EITC exposure (ages 0–49)	0.979	0.975	0.985***	0.985***	0.984**	0.977	0.978
	(0.001)	(0.001)	(0.001)	(0.001)	(0.002)	(0.001)	(0.002)
Observations	5260	3233	2027	1325	702	3599	850

*Note:* Columns 2 & 3 split the full sample (column 1) into individuals reporting any work disability between ages 50 and 61 and those who never report work disability. Columns 4 & 5 split the sample of individuals reporting any work disability between ages 50 and 61 into those who experience non‐chronic or non‐severe work disability and those who experience chronic and severe work disability. The final two columns split the sample into those who are likely to receive DI and those who likely do not. “nH” refers to non‐Hispanic, “HS” refers to High School, “HS/GED” refers to High School or General Equivalency Diploma, “College” refers to reports of at least 4 or more years of college. Income refers to total reported household income in 2020 dollars. Overall Health refers to the average of self‐reported health on a 5 point Likert scale with Excellent = 97.5, Very Good = 90, Good = 75, Fair = 50, and Poor = 15 (Erickson 1998; Erickson et al. 1995; Halliday et al. 2021). ADL refers to Activities of Daily Living. + *p* < 0.1.

^*^ *p* < 0.05.

^**^ *p* < 0.01.

^***^ *p* < 0.001.

*Source:* Authors' calculations using PSID public data.

These patterns are largely amplified when we consider the severity of reported work disability. For example, 10.0% of the non‐chronic/non‐severe work‐limitation group has less than a high school credential, while 21.1% of the chronic and severe work‐limitation group does. Average income and self‐reported health for those with non‐chronic or non‐severe limitations are markedly higher relative to persons with chronic and severe limitations. ADLs are also much more common among those with chronic and severe work limitations. The sample partition based on probable DI receipt echoes the described patterns for self‐reported work disability. Individuals likely to have DI awards tend to have lower educational attainment, lower income, poorer health, and more time living with ADL limitations.

Table 1 also reports the maximum number of EITC‐eligible dependent children in adulthood, and this measure is very similar across work limitation status. Additionally, Table 1 shows the share of lifetime exposure accounted for by the *federal* EITC program. This share is uniformly high, about 98% overall, and is only slightly higher for those who ever report a work limitation than for those who never do (0.985 vs. 0.975), showing that cross‐group differences in cumulative exposure are not primarily driven by differential state supplements.

Importantly, we observe a negative correlation between total EITC exposure (measured in dollars) and later‐life work disability. Individuals without work disability between ages 50 and 61 were exposed to an average of approximately $59,535 in EITC benefits over ages 0–49, while those with any reported work disability were only exposed to roughly $44,459—a statistically significant difference of about $15,000, or about 25% fewer EITC dollars. Individuals with non‐chronic or non‐severe limitations between ages 50 and 61 were exposed to more EITC dollars before age 50 compared to their peers with a chronic and severe work disability, although the difference is smaller in magnitude. A similar pattern is observed when comparing DI awardees with non‐awardees; the average DI awardee was exposed to around $6400 fewer EITC dollars before age 50. Splitting EITC exposure into childhood (i.e., ages 0–18) and adulthood (i.e., ages 19–49) reveals very similar patterns, with one unexpected exception where childhood EITC exposure for those with chronic and severe limitations was actually higher relative to individuals with non‐chronic or non‐severe limitations, although the difference remains small in magnitude.

Note that the much larger cumulative EITC exposure in ages 19–49 compared to exposure in childhood is expected for our cohorts and reflects policy timing and life‐cycle family formation rather than a data issue. The EITC started in 1975, and real generosity remained modest until the major federal expansions in 1986, 1990, and 1993 (with an additional tier in 2009). Many state supplements were adopted mainly in the 1990s–2000s. For the 1933–1969 birth cohorts, this means little or no exposure during childhood but substantial exposure in adulthood, precisely when they have their own qualifying children and thus larger statutory credits. Consequently, cumulative exposure mechanically accrues far more in ages 19–49 than in ages 0–18 for these cohorts (see EITC history in Section 2).

**FIGURE 1:**
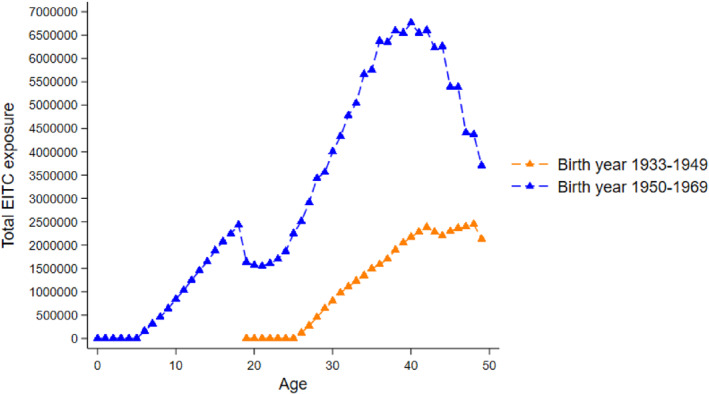
Total Potential EITC Exposure by Age for Two Birth‐Cohort Groups. *Source:* Authors' calculations using PSID public data. Points show the maximum potential EITC dollars (2020 USD) by age, separately for birth cohorts 1933–1949 (orange, dashed) and 1950–1969 (blue, dashed). The maximum potential EITC dollars equal the statutory maximum federal credit plus any state supplement, determined by each person‐year's state of residence and number of EITC‐qualifying children. The profiles are descriptive and illustrate that exposure is near‐zero in childhood for earlier cohorts and peaks in adulthood, with substantially larger adult exposure for later cohorts who became parents during the major federal expansions and the spread of state credits.

To further illustrate this point, Figure 1 plots total potential EITC exposure (2020 USD) by age for two birth‐cohort groups: 1933–1949 and 1950–1969. For each age, we sum across the statutory maximum federal credit plus any state supplement implied by each person's state of residence and EITC‐qualifying child count in that year. The series therefore visualize policy‐driven generosity at each age of the life cycle for two groups of birth cohorts in our analytic sample. Note this figure is a descriptive display of the policy environment.

Three observations emerge. First, exposure during childhood is minimal with low variation for these cohorts, whereas exposure accumulates in adulthood with a clear peak in the late 30s/early 40s, mirroring family formation and coinciding with large federal expansions and the diffusion of state supplements. Second, the later/younger cohorts who reached parenthood during the expansionary 1990s/2000s exhibit much higher adult exposure than the earlier/older cohorts. Third, the local dip in early 20s for later cohorts is consistent with aging out of child eligibility before respondents form their own EITC‐eligible households. Together, these patterns are consistent with the difference in EITC exposure for “0–18 vs. 19–49” shown in Table 1: for the 1933–1969 cohorts, the policy's start in 1975 and the major generosity increases in adulthood years imply that most cumulative exposure for our sample accrues after age 18, not during childhood. This figure therefore also previews why our adult‐exposure coefficients are estimated more precisely than childhood coefficients: the key exposure measure simply has far more variation in adulthood.

## 5 Results

**FIGURE 2:**
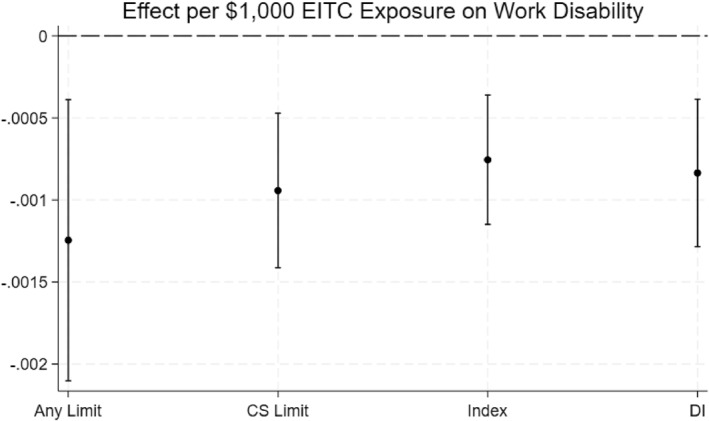
Main Effects of $1000 of EITC Exposure on Work Disability. *Source:* Authors' calculations using PSID public data. Full regression results are in Appendix Table A1. “Any Limit” is a binary dependent variable for any reported work limitation between ages 50 and 61. “CS Limit” is a binary characteristic for reported chronic and severe work limitations between ages 50 and 61. “Index” is the continuous work limitation index. “DI” is a binary variable for whether the individual likely had DI before age 65. All results are from an OLS regression model outlined in the methods section. 95% confidence intervals are illustrated.

The descriptive statistics discussed above suggest that the EITC is negatively correlated with work disability, as hypothesized. Turning to our empirical specification that supports a more causal interpretation, we find strong evidence that the EITC exposure significantly and meaningfully reduces the probability of work disability. Figure 2 illustrates the effect of exposure to an additional $1000 of EITC on our four measures of work disability observed between ages 50 and 61: ever experiencing a work disability, having a chronic and severe work disability, the work limitation index, and DI receipt. Although the individual effect sizes are modest (see Appendix Table A1), they accumulate into sizable overall effects. For example, $10,000 of cumulative EITC exposure would translate into a 1.25 percentage‐point drop in the likelihood of experiencing any work disability, a 0.94 percentage‐point reduction in experiencing a chronic and severe work disability, and a 0.84 percentage‐point decrease in the likelihood of DI receipt. Additionally, each $10,000 of EITC exposure reduces the predicted work limitation index by approximately 0.0076, which when scaled to a $50,000 additional exposure would mean one less mild work limitation report for every 10 reports over ages 50–61. Note that we translate per‐$1000 coefficients into per‐$10,000 effects just for readability; these are marginal changes along the continuous EITC exposure measure rather than $0‐vs‐$10,000 comparisons.

### 5.1 Heterogeneity by Race/Ethnicity, Sex, and Educational Attainment

**FIGURE 3:**
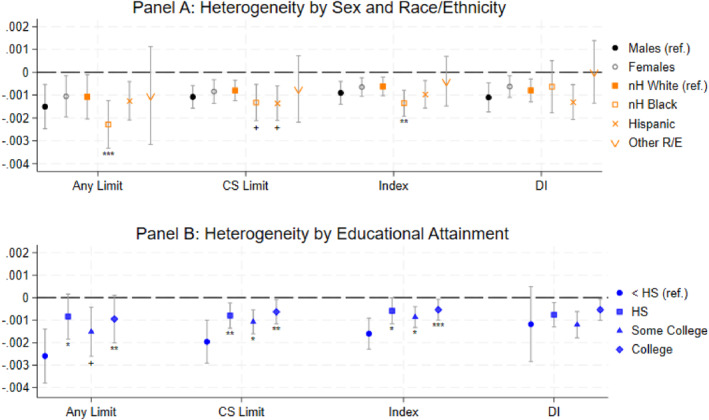
Heterogeneous Effects of $1000 of EITC Exposure on Work Disability by Race/Ethnicity, Sex, and Educational Attainment. *Source:* Authors' calculations using PSID public data. Full regression results are in Appendix Table A2. “Any Limit” is a binary dependent variable for any reported work limitation between ages 50 and 61. “CS Limit” is a binary characteristic for reported chronic and severe work limitations between ages 50 and 61. “Index” is the continuous work limitation index. “DI” is a binary variable for whether the individual likely had DI before age 65. All results are from an OLS regression model outlined in the methods section. 95% confidence intervals are illustrated. + *p* < 0.1, *p < 0.05, **p < 0.01, ***p < 0.001 relative to identified reference category (i.e., males, non‐Hispanic White, and less than high school educational attainment).

Our analysis reveals that the EITC's mitigating effects on work disability extend across most demographic groups (Figure 3). The only exception is a positive effect of the EITC on DI awards for “other” races/ethnicities; however, the wide confidence interval suggests that sizable negative effects cannot be ruled out. While males appear to benefit more from EITC exposure compared to females, this difference is not statistically significant for any measure of work disability. Non‐Hispanic Black Americans consistently benefit more from EITC exposure than their non‐Hispanic White counterparts, except for DI awards. Specifically, for each additional $10,000 of EITC exposure, non‐Hispanic Black Americans see an additional 1.2 percentage‐point reduction in the likelihood of experiencing any work disability and an additional 0.5 percentage‐point decrease in the likelihood of experiencing a chronic and severe work disability compared to non‐Hispanic White individuals. These findings imply that the EITC may play a role in narrowing the existing disparities in self‐reported work disability observed between these groups (Figure 3, panel A and Appendix Table A2).

Furthermore, when using educational attainment as an indicator of socio‐economic status (SES), we also find evidence of markedly stronger effects of the EITC among low‐SES populations (Figure 3, panel B). Individuals with less than a high school credential consistently experience the strongest EITC effects across all self‐reported work disability measures. This pattern is consistent with the EITC's intended targeting of lower‐ and medium‐income workers. Importantly, among those with higher education, the effect of the EITC on work disability prevalence is smaller, serving as a placebo test where we expect null results among individuals who are less likely to receive the tax credit due to their higher income. This set of results is consistent with the EITC literature, which has been using education as a proxy for EITC eligibility or an indicator for the likelihood of receiving EITC benefits.

### 5.2 Heterogeneity by Age at Exposure

Our measure of EITC exposure spans 5 decades of the life course. Of particular importance to policymakers, though, is determining when exposure matters most. While a significant body of literature emphasizes the importance of childhood exposure to the social safety net for later life outcomes, including health outcomes (Bastian and Michelmore 2018; Braga et al. 2020; Hoynes et al. 2016; Miller and Wherry 2019), evidence also points to significant health improvements from adult EITC exposure (Evans and Garthwaite 2014; Lenhart 2019). Our estimates suggest that effects could be driven by adulthood exposure (Table 2); however, childhood exposure has larger, but imprecisely measured, estimates.^14^ This suggests that while not pinpointing the most crucial life phase for exposure, our results convincingly indicate that exposure in adulthood plays a vital role, even after controlling for childhood exposure.

**TABLE 2:** The effect of the EITC on work disability.

	(1)	(2)	(3)	(4)
	Any limit Ages 50–61	CS limit Ages 50–61	Index Ages 50–61	DI
EITC (ages 0–49)	−0.00125**	−0.000943***	−0.000755***	−0.000835***
	(0.000423)	(0.000233)	(0.000195)	(0.000222)
Childhood EITC (ages 0–18)	−0.0318	−0.0130	−0.0149	0.00509
	(0.0468)	(0.0207)	(0.0255)	(0.0269)
Adulthood EITC (ages 19–49)	−0.00122**	−0.000931***	−0.000742***	−0.000841***
	(0.000423)	(0.000232)	(0.000188)	(0.000227)
Observations	5260	5260	5260	4449

*Note:* “Any Limit” is a binary dependent variable for any reported work limitation between ages 50 and 61. “CS Limit” is a binary characteristic for reported chronic and severe work limitations between ages 50 and 61. “Index” is the continuous work limitation index. “DI” is a binary variable for whether the individual likely had DI before age 65. All results are from an OLS regression model outlined in the methods section. The first row (EITC ages 0—49) is from one regression, while the second and third rows (Childhood and Adulthood EITC) are included together in a separate regression. + *p* < 0.1.

*p < 0.05.

^**^ *p* < 0.01.

^***^ *p* < 0.001.

*Source:* Authors' calculations using public PSID data.

### 5.3 Age Trajectories of Work Disability

**FIGURE 4:**
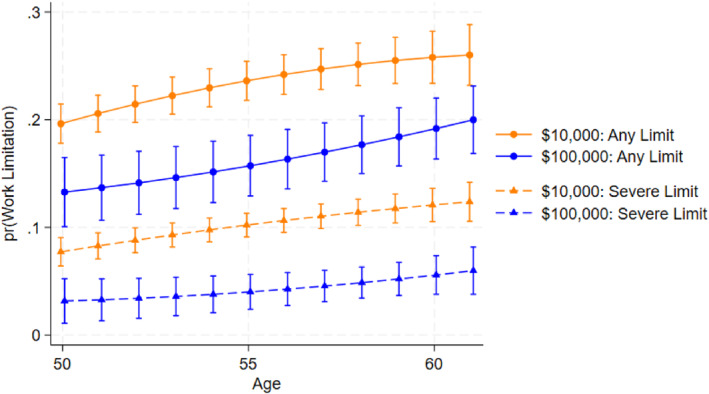
EITC effects on Work Disability between ages 50 and 61 with person‐wave observations. *Source:* Authors' calculations using PSID public data. Results are from an OLS regression (linear probability model) of self‐reported work disability (any limitation or severe limitations) at each wave the individual is between ages 50 and 61. Regressions include demographic controls, individual controls, environment controls, birth state and cohort fixed effects analogous to the main specification. Ninety‐five percent confidence intervals are illustrated.

**FIGURE 5:**
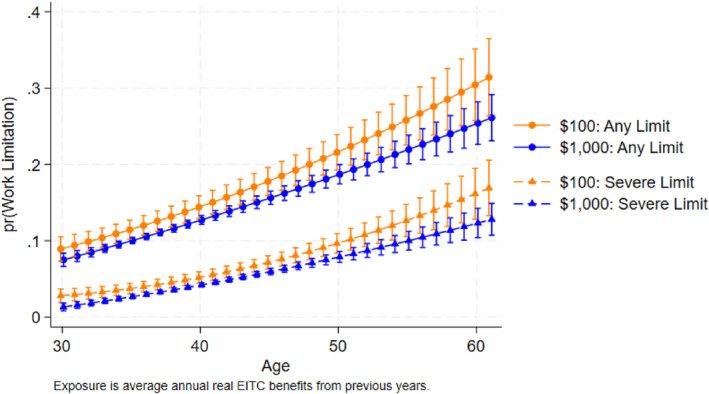
EITC effects on Work Disability between ages 30 and 61 with person‐wave observations. *Source:* Authors' calculations using PSID public data. EITC exposure is measured as average annual previous exposure up to age minus one. Results are from an OLS regression (linear probability model) of self‐reported work disability (any limitation or severe limitations specifically) at each wave the individual is between ages 30 and 61. Regressions include demographic controls, individual controls, environment controls, birth state and cohort fixed effects analogous to the main specification. Ninety‐five percent confidence intervals are illustrated.

Aggregating work disability from age 50 to 61 could mask how prevalence evolves with age. To visualize these dynamics, we plot, in Figures 4 and 5, the predicted probability for work disability (and severe disability) over an age profile using regressions with person‐by‐wave outcomes as opposed to the main person‐level model. This specification is similar to that employed by Hoynes et al. (2016).

For Figure 4, we estimate linear‐probability models with each person contributing multiple observations between ages 50 and 61, inclusive. The dependent variable is an indicator for reporting any work limitation (or, in a separate specification, a severe work limitation). Key regressors include a person's life‐course cumulative EITC exposure (from birth through age 49, expressed in thousands of 2020 dollars), a flexible function of age (age and age‐squared), and interactions between EITC exposure and age to allow EITC effects to evolve along specific age trajectories. The control set mirrors the main model, but with time‐varying marital and poverty status measured at each wave an individual reports work disability rather than life course aggregates. We also include birth‐state, birth‐cohort fixed effects, and apply PSID weights. Using the fitted models, we generate predicted probabilities of work disability by age for two illustrative EITC exposure scenarios, relatively low ($10,000) and relatively high ($100,000) lifetime exposure, and plot these predictions with 95% confidence intervals. Across ages 50–61, the high‐exposure group exhibits lower prevalence of both any work disability and any severe work disability.

For Figure 5, we extend the person‐wave approach to also examine the evolution of work disability earlier in the life course (ages 30–61). The main difference is that the lifetime total EITC exposure variable (ages 0—49) is replaced with a time‐varying measure of average annual EITC exposure accumulated up to the current age minus one to reflect changes in exposure over time while also preserving temporal ordering of the exposure and outcome of interest. The same controls, fixed effects, and weights are used. We report predicted probabilities of work disability for low ($100) versus high ($1000) average annual exposure to provide interpretable benchmarks. The two series are separated by a small level difference through the 30s and early 40s, with substantial overlap of the 95% confidence intervals; that is, trajectories are essentially parallel until mid‐life. By the late 50s, however, the gap appears to grow, although confidence intervals in the estimates continue to overlap. This pattern suggests one pathway by which the EITC may fend off work disability is by quelling the effects of advancing age on the likelihood of acquiring a work disability.

### 5.4 Robustness

We next show the results of several robustness checks to bolster confidence in our conclusions. We organize these robustness checks around five questions: identification, timing, sample scope, inference, and specification.

#### 5.4.1 Identification and Sources of Variation

First, to address the potential endogeneity of state EITC policies with state economic conditions and co‐occurring policies, we re‐estimate the models using only the federal EITC exposure. Coefficient estimates are virtually unchanged across all four outcomes (Table 3, row 2). The stability of the coefficient under the federal‐only measure should not be read as evidence that state EITCs have no impact. Rather, given the timing and scale relevant to our 1933–1969 birth cohorts, federal reforms provided sufficient identifying variation on their own. As noted earlier, state EITCs are typically percentages of the federal credit and were adopted gradually. For many cohort‐by‐age combinations in our sample, federal changes dominate the life‐course exposure, while state supplements arrive later and at a smaller scale. Dropping the state portion thus removes little independent variation, leaving the estimated effect essentially unchanged. This federal‐only result is best viewed as an identification robustness: our main conclusions do not hinge on potentially endogenous cross‐state policy differences. It remains entirely plausible, and consistent with prior evidence summarized in Section 2, that state EITC enhancements improve health and well‐being where they are salient; our cohorts simply accrued most of their lifetime EITC exposure from federal law changes.

**TABLE 3:** Robustness of main results.

	N: Work limit	N: DI	Any limit ages 50–61	CS limit ages 50–61	Index Ages50‐61	DI
(1) Main results	5260	4449	−0.00125**	−0.00094***	−0.00076***	−0.00084***
			(0.00042)	(0.00023)	(0.00019)	(0.00022)
(2) Federal EITC only	5260	4449	−0.00127**	−0.00109***	−0.00081***	−0.00092***
			(0.00040)	(0.00024)	(0.00021)	(0.00024)
(3) Non‐movers	3487	2820	−0.00100+	−0.00088*	−0.00074*	−0.00096*
			(0.00054)	(0.00042)	(0.00035)	(0.00036)
(4) State‐time trends	5260		−0.00127**	−0.00095***	−0.00075***	−0.00087***
			(0.00041)	(0.00022)	(0.00018)	(0.00021)
(5) Exclude those with early work limitation	3142	2690	−0.00060	−0.00000	−0.00009	−0.00040
			(0.00045)	(0.00025)	(0.00014)	(0.00034)
(6) Exclude those with early severe limitation	4312	3699	−0.00131*	−0.00065**	−0.00060**	−0.00073**
	4312	3699	(0.00052)	(0.00022)	(0.00020)	(0.00027)
(7) Drop SEO	3040	2783	−0.00094+	−0.00093**	−0.00061**	−0.00081**
			(0.00055)	(0.00026)	(0.00022)	(0.00027)
(8) Unweighted	5260	4449	−0.00137***	−0.00089**	−0.00081***	−0.00059**
			(0.00036)	(0.00025)	(0.00019)	(0.00021)
(9) Add 1923–1932 cohorts	5516	4449	−0.00123*	−0.00089**	−0.00074***	−0.00084***
			(0.00045)	(0.00025)	(0.00020)	(0.00022)
(10) Add 2021 data	5260		−0.00118**	−0.00090***	−0.00072***	
	5260		(0.00040)	(0.00022)	(0.00018)	
(11) Add 1970–1971 cohorts	5494	4683	−0.00121**	−0.00084***	−0.00073**	−0.00078***
	5494	4683	(0.00040)	(0.00023)	(0.00022)	(0.00020)
(12) Remove 1959 ‐ 1969 cohorts	3618	2814	−0.00153*	−0.00131*	−0.00071*	−0.00154**
	3618	2814	(0.00062)	(0.00051)	(0.00029)	(0.00050)
(13) PSID survey design	5260	4449	−0.00125***	−0.00094***	−0.00076***	−0.00084***
	5260	4449	(0.00035)	(0.00025)	(0.00018)	(0.00024)
(14) No clustering	5260	4449	−0.00125**	−0.00094***	−0.00076**	−0.00084*
	5260	4449	(0.00040)	(0.00028)	(0.00023)	(0.00033)
(15) Remove F.E. for # EITC kids in adulthood	5260	4449	−0.00124**	−0.00094***	−0.00075***	−0.00084***
	5260	4449	(0.00042)	(0.00023)	(0.00020)	(0.00022)
(16) Ever married	4416	3721	−0.00114*	−0.00079**	−0.00074**	−0.00091***
			(0.00052)	(0.00024)	(0.00022)	(0.00024)
(17) Never married	844	728	−0.00122	−0.00170+	−0.00103+	−0.00052
			(0.00119)	(0.00091)	(0.00057)	(0.00099)
(18) EITC children	4788	4036	−0.00131*	−0.00103***	−0.00083***	−0.00074**
			(0.00049)	(0.00028)	(0.00023)	(0.00024)
(19) No EITC children	472	413	−0.00672	−0.00369	−0.00204	0.00307
			(0.00895)	(0.00898)	(0.00503)	(0.00868)

*Note:* All coefficients are from a separate regression capturing $\beta_{1}$ in Equation 1. “Any Limit” is a binary dependent variable for any reported work limitation between ages 50 and 61. “CS Limit” is a binary characteristic for reported chronic and severe work limitations between ages 50 and 61. “Index” is the continuous work limitation index. “DI” is a binary variable for whether the individual likely had DI before age 65. + *p* < 0.1.

^*^ *p* < 0.05.

^**^ *p* < 0.01.

^***^ *p* < 0.001.

*Source:* Authors' calculations using public PSID data.

Second, row 3 of Table 3 demonstrates that although we have a significantly smaller sample after removing those who moved across state lines before age 50, which is our way to address the potential concern that individuals eligible for the EITC and other safety‐net measures may migrate to states with more generous policies, the coefficient estimates are very close to our main estimates and remain statistically significant. Third, replacing state fixed effects with a state‐specific time trend does not change our findings (row 4, Table 3).

#### 5.4.2 Temporal Ordering

There could be concern with the concurrent measurement of EITC exposure and work disability, specifically for individuals acquiring work disability prior to age 50. Our baseline design already accumulates exposure through age 49 and then measures work‐limitation outcomes at ages 50–61, but some individuals report limitations earlier in life (including “always limited” at first interview), for whom onset precedes our outcome window. To enforce temporal ordering mechanically, in rows 5 and 6 of Table 3, we exclude individuals who (a) reported any work disability before age 50, or (b) reported having a severe work disability before age 50. As expected, these restrictions reduce sample size and statistical power. Under the stricter “any work disability pre‐50 limit” exclusion, coefficients remain negative but imprecisely estimated. Under the “severe pre‐50 limit” exclusion, estimates remain negative and statistically significant. Taken together, these results indicate our main conclusions are not driven by early‐onset cases or by conflating exposure with pre‐existing disability, thereby directly alleviating any concern about onset timing for work limitations in the PSID.

#### 5.4.3 Sample Design

**FIGURE A1:**
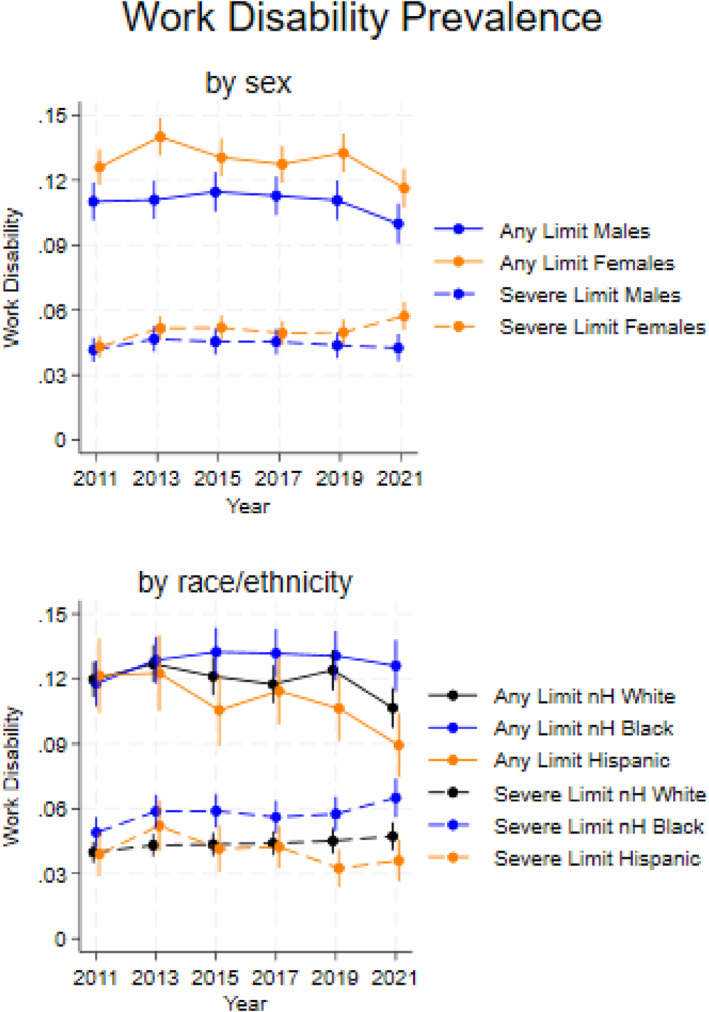
Psid work disability reports 2011—2021. *Source:* Authors' calculations using PSID public data. 95% confidence intervals are illustrated.

Excluding the Survey of Economic Opportunity (SEO) from the sample (row 7), or unweighting our sample (row 8), to address the potential bias that may arise from the original PSID sampling design, yields consistent results. Adding in the 1923‐1932 birth cohorts (row 9) that were never exposed to state‐varying EITC policy only changes our estimates slightly. We have also omitted 2021 data in the main analysis since COVID‐19 pandemic's disruptions to labor market participation could have affected work disability reports. In Appendix Figure A1, we illustrate that for some demographic groups, there is an unexpected decrease in the prevalence of work disability reports in the 2021 wave, accompanied by an increase in severe reports. Nevertheless, utilizing 2021 data on self‐reported work disability (row 10) does not alter our main conclusions regarding the effect of the EITC on work disability, nor does incorporating two additional birth cohorts (row 11), the 1970 and 1971 birth cohorts, who are observed at ages 50 and 51 in 2021. Finally, to address the unbalanced panel that arises because later‐born cohorts are observed in fewer waves (annual through 1997; biennial thereafter), we re‐estimate the models excluding the 1959–1969 birth cohorts (row 12), so that all retained cohorts can be followed through age 61 by 2019. This restriction equalizes follow‐up across cohorts in the outcome window and removes the youngest groups that are observed for less time leading up to retirement. Estimates remain similar in sign and magnitude to the main results across all outcomes, indicating that our conclusions are not driven by shorter follow‐up for the youngest cohorts or sample design more generally.

#### 5.4.4 Inference

We also assess the sensitivity to how inference is conducted. First, we incorporate the PSID survey‐design specifications (stratification and clustering) while using the same weights as the main models. Second, we report non‐clustered standard errors as an alternative. These results are reported in rows 13 and 14 of Table 3. Estimates are stable across these variants, and our main results (with state‐clustered standard errors) are the most conservative for inference.

#### 5.4.5 Specification and Subgroup Diagnostics

Results are also stable when omitting the fixed effect for the maximum number of EITC‐eligible children in the adult household (Table 3, row 15). Additionally, we anticipate that effects may vary by marital status. We find effects predominantly concentrated on individuals who were ever married, although the sample for never married individuals is considerably smaller and, correspondingly, imprecisely estimated (rows 16 and 17). Finally, when we split the sample by whether the individual ever had EITC‐eligible children, as expected, effects are concentrated among those with children (ample exposure variation), while estimates for the subgroup without EITC‐eligible children are imprecise given the much smaller sample size and minimal exposure under the childless EITC (rows 18 and 19). Taken together, the evidence supports a common conclusion: effects are identified from within‐family‐size policy variation, dominated by federal changes for these cohorts, and are robust to migration, sample design, cohort bounds, and alternative inference.

### 5.5 Severe and Objective Health Outcomes

To understand whether our main results extend to more objective, serious health conditions, we analyze two additional outcomes. First, we construct an indicator for respondents ever experiencing any difficulty with Activities of Daily Living (ADLs) before age 62. ADLs index basic self‐care (e.g., dressing, bathing) and are widely interpreted as markers of serious functional loss. Second, we define an indicator for ever reporting a doctor‐diagnosed physical health condition which the respondent indicates limits their normal daily activities before age 62. This “limiting condition” measure anchors self‐reports to both a medical diagnosis and explicit functional consequences. By design, both measures mitigate concerns that our findings are driven solely by subjective perceptions of “work limitation.” They also help corroborate whether policy‐induced health improvements translate into meaningful decrements in morbidity.

For both outcomes, we use the same specification as the main analysis (Equation 1), measuring cumulative EITC exposure in thousands of 2020 USD. Estimates (see Table 4) are directly comparable to our main results. Specifically, greater life‐course EITC exposure is linked with a lower and statistically significant incidence of both severe and objective outcomes. The estimated coefficients imply that an additional $10,000 of EITC exposure corresponds to 0.95 percentage‐point lower risk of ever experiencing an ADL difficulty and 0.73 percentage‐point lower risk of ever having a limiting doctor‐diagnosed condition prior to age 62. These effect sizes are similar in magnitude to our main estimates for self‐reported work limitation (Table 2), reinforcing that the policy‐health relationship we document is not confined to an individual's subjective self‐assessment of work disability. Rather, it extends to outcomes that require either impaired basic functioning (ADLs) or a physician‐diagnosed functional limitation.

**TABLE 4:** Severe and objective health outcomes.

	N	Ever ADL before age 62	Ever limiting physical health before age 62
Estimates	5260	−0.00095**	−0.00073*
S.E.		(0.00033)	(0.00035)

*Note:* All coefficients are from a separate regression capturing $\beta_{1}$ in Equation 1. “Ever ADL before Age 62” is a binary dependent variable for any ADL before age 62. “Ever Limiting Physical Health before Age 62” is a binary characteristic for ever reporting limiting physical health condition before age 62. + *p* < 0.1.

^*^ *p* < 0.05.

^**^ *p* < 0.01.

***p < 0.001.

*Source:* Authors' calculations using public PSID data.

### 5.6 Discussion: Mechanisms Underlying the Impact of EITC on Work Disability

The findings of our study, which demonstrate a statistically significant reduction in work disability with increases in maximum EITC benefits to which individuals are exposed, prompt an exploration of the underlying mechanisms driving this relationship. This section delves into a few mechanisms that could potentially explain our findings, informed by the EITC literature and consistent with the theoretical motivation grounded in Grossman's health capital model.

A key starting point is that the EITC, as a pivotal element of the U.S. social safety net, has been consistently associated with increased labor market participation, especially for single mothers, which is linked to an immediate increase in earnings and thus provides an economic buffer for low‐income households (Bastian 2020; Bastian and Michelmore 2018; Chetty et al. 2013; Dickert et al. 1995; Eissa and Hoynes 2006; Eissa and Liebman 1996; Grogger 2003; Hoynes 2009; Hoynes and Patel 2018; Meyer 2010; Meyer and Rosenbaum 2001). The increased labor market participation and income due to the EITC can also influence many aspects of the recipients' and their families' lives, including access to health resources (Eissa and Hoynes 2006; Hoynes 2009), health behaviors (Averett and Wang 2013; Cowan and Tefft 2012), education (Bastian and Michelmore 2018; Chetty et al. 2011; Dahl and Lochner 2012), marriage and child‐bearing patterns (Dickert‐Conlin and Houser 2002; Michelmore and Lopoo 2021), crime rates (Lenhart 2021), and employment opportunities and workplace environments (Dickert et al. 1995).

According to the Grossman model (see Section 3), these direct and indirect effects of the EITC could collectively exert a substantial impact on recipients' and their family members' health, by affecting the health production function and the budget constraints. Increased employment itself could contribute to an individual's sense of purpose and belonging, factors known to positively affect mental health (Jahoda 1981; Zechmann and Paul 2019). Increased financial support attributable to the EITC alleviates the immediate economic stresses facing these households, directly influencing health outcomes by reducing the mental and physical stress associated with financial insecurity (Ettner 1996; Gardner and Oswald 2007; Golberstein 2015). Furthermore, studies have shown that additional EITC benefits lead to improved birth outcomes (Hoynes et al. 2015; Markowitz et al. 2017; Strully et al. 2010), an increased likelihood of reporting very good or excellent health and a decreased likelihood of obesity in young adulthood (Braga et al. 2020), and improved health outcomes as adults (Bastian 2020; Cowan and Tefft 2012; Evans and Garthwaite 2014; Gangopadhyaya et al. 2020; Lenhart 2019; Muennig et al. 2016). These improvements in health at early‐ and mid‐life stages can lay the foundation for better health in later years, thereby reducing the likelihood of work disability.

It is important to acknowledge that the EITC might induce individuals to take on more physically demanding jobs, potentially increasing the risk of work disability. This scenario would mean our estimates are a lower bound of the EITC's true effect. Conversely, the EITC may also encourage individuals to work fewer hours or seek less physically demanding jobs (possibly with higher pay) to maximize EITC benefits. This latter possibility could be considered one potential mechanism for our finding that EITC exposure is associated with a reduced likelihood of work disability. These divergent effects suggest that the actual impact of EITC on work disability could be more complicated than our findings indicate, thus our results are conservative estimates of the EITC's effect on work disability.

In summary, our findings on the EITC's impact on reducing work disability can be attributed to a multifaceted mechanism involving enhanced labor market attachment, economic buffering, increased access to health resources, and improved quality of life in various aspects. These mechanisms collectively contribute to better health outcomes and a reduced likelihood of work disability among EITC recipients, as evidenced by our findings.

### 5.7 Quantifying the Economic Impacts of Increased EITC Exposure on DI Awards

To quantify the economic impacts of increased EITC exposure on future DI awards, we start with estimating the cost of a DI award per awardee. The average age of a DI applicant is 46.6 years old (Maestas et al. 2021). Just over half of initial applications (54%) result in a DI award (Maestas et al. 2021); however, older applicants are more likely to receive an award based on administration rules for more lenient decisions at ages 50 and 55 (Deshpande et al. 2019). Drawing from this evidence, we first assume the average age of DI award initiation to be around 51. Second, in estimating the age at DI termination, we acknowledge that the DI program is typically an absorbing state. Among terminations in 2021, 54.6% were due to reaching the full retirement age (and benefits converting to retirement benefits), and 33.6% were due to the awardee's death prior to the full retirement age (Social Security Administration 2022, Table 50). We assume awardees who pass away or are terminated for other reasons (i.e., the remaining 12% in 2021 estimates) do so about halfway between award initiation and full retirement age (e.g., age 59 with a full retirement age of 67). Thus, roughly 55% of awardees receive DI for 16 years, while 45% receive DI for 8 years. With an average monthly benefit of $1358.30 in 2021 (Social Security Administration 2022) the projected lifetime DI benefit in 2021 dollars would be (0.55×($1,358.30×12×16))+(0.45×($1,358.30×12×8))=$202,115.04. In 2020 dollars, the currency of our results, this amounts to approximately $193,046 =$202,115.041.047.

Our estimates indicate that each $1000 increment in EITC exposure decreases the likelihood of a DI award by 0.0835 percentage points. Considering a population of 1 million individuals, this would amount to 835 fewer DI awards valued at over $161 million (= 835*$193,046) per $1 billion of EITC exposure ($1000 x 1 million people). Naturally, exposure represents the selected policy parameters, but it does not equate with policy expenditures. Eligible households must meet specific work and earnings requirements ‐ only about 20% of U.S. households qualify (IRS, 2023b). Additionally, in recent years, just under 80% of eligible households claim the EITC credit (IRS, 2023a). For simplicity, if we assume household sizes are similar across EITC‐eligible and non‐EITC‐eligible households, $1 billion in EITC exposure roughly reflects $160 million spent on the EITC program (=$1billion×20%eligible×80%take−up).

As a final step, we adjust for the timing of costs and expected savings. If we take the midpoint of our exposure interval (age 25), there are about 26–42 years between exposure and when savings are anticipated to accrue. Assuming benefits (in terms of savings from fewer DI awards) accrue 34 years after EITC expenses (the midpoint of our range of 26–42 years between EITC exposure and expected savings from averted DI awards) with an average annual rate of return of 7% (Siegel 2021, page 35), the present value of the $161 million in averted DI awards is about $16.14 million, or roughly 10% of the $160 million in estimated EITC expenditures. Therefore, although savings from the averted DI program are not expected to cover the entirety of the increases in EITC generosity, we project a substantial offset.

## 6 Discussion and Conclusion

This study examines the effects of individuals' exposure to EITC on work disability and DI in late adulthood. Using PSID data and exogenous variations in EITC policies, we find that EITC decreases the prevalence of work disability reports and DI awards, as hypothesized. Thus, to answer our first research question, the EITC may be viewed as a public health investment tool to bolster health capital and reduce future healthcare and social security costs.

Our analysis also reveals intriguing patterns of heterogeneity across various demographic subgroups, answering our second research question. Specifically, EITC exposure appears most influential in decreasing the prevalence of work disability among non‐Hispanic Black Americans relative to non‐Hispanic White Americans. This could relate to different efficiencies in translating EITC exposure into improved health outcomes, constraints, and/or initial health endowments. Furthermore, using educational attainment as a proxy for SES, we find stronger effects of EITC in low‐SES populations, which suggests the EITC program is achieving its targeting goals. Future policy could focus on optimizing EITC parameters to further reach these vulnerable populations.

Of particular note, our results suggest that EITC exposures beyond childhood offer protection against work disability in later years. Coefficient estimates on childhood exposure, however, are larger in magnitude than those for adulthood exposure across all examined outcomes. Considering the EITC's inception in 1975, our cohorts of study (1933–1969) experienced relatively little childhood exposure, with even the youngest cohort (1969) exposed to zero EITC from ages 0–5. Only the 1957–1969 cohorts had any exposure during childhood, and the 1957–1965 cohorts were exposed only to Federal EITC benefits. Typically, the onset of work limitation occurs later in life. Therefore, we hypothesize that in order to adequately answer our third research question regarding which life stage is EITC exposure most effective in preserving health capital, future research is warranted. However, we have to consider the possibility that the required data to fully understand the relative importance of childhood versus adulthood exposure might not yet be available. As birth cohorts from the 1970s and 1980s age into retirement, it would provide a better opportunity to revisit this important question. It is clear, however, that the EITC is influential for the prevalence of work disability. Higher EITC benefits, even during adulthood, can reduce the incidence of work disability and the need for DI.

Evidence on EITC participation dynamics indicates that benefit receipt is generally episodic rather than continuous for many families. For example, Dowd and Horowitz (2011) document (i) that a majority of EITC claimants receive the credit for only one or 2 years, (ii) comparatively few claimants sustain benefits for long spells, and (iii) that re‐entry (churn) is common as circumstances change. Our empirical strategy does not depend on which receipt pattern an individual experiences: we measure cumulative life course exposure (in thousands of 2020 USD) up to age 49 to explain different work disability outcomes after age 50. The fact that many families' benefit receipt pattern is short or episodic suggests that our exposure measure likely attenuates per‐dollar effects, making our estimates conservative. The finding that greater cumulative exposure is nonetheless associated with meaningfully lower risks of work disability and DI suggests that intermittent boosts, especially when they coincide with periods of high health‐production returns, can yield lasting improvements in functional capacity.

We also estimate that approximately 10 percent of current EITC outlays could be offset by anticipated reductions in work disability, specifically DI awards, in the future. This finding complements recent work by Bastian and Jones (2021) who estimate that the EITC has an 83% 1‐year self‐financing rate. Part of their calculation includes a concurrent 0.1% point reduction in the likelihood of receiving a DI or Supplemental Security Income award for every $1000 increase in exposure to Federal EITC benefits. Our focus is, however, on the long‐term effects that Bastian and Jones (2021) importantly acknowledge would likely enhance the self‐financing capabilities of the EITC program. We provide empirical estimates of one specific pathway that could cover a portion of the remaining 17% 1‐year shortfall of self‐financing costs of the EITC program from their estimates.

The policy implications of this study are both timely and significant, given the ongoing debates surrounding social safety net reforms. Our findings unequivocally demonstrate that exposure to the EITC has substantive effects on reducing work disability in later life. More pointedly, the differential benefits accrued by demographic subgroups, particularly among non‐Hispanic Black Americans, suggest that the EITC could serve as a strategic lever to alleviate existing health disparities. As policymakers grapple with the complexities of healthcare costs and social inequality, the EITC emerges from our analysis as an effective, multi‐faceted intervention deserving of serious consideration in future legislative agendas.

## Conflicts of Interest

The research reported herein was derived in whole or in part from research activities performed pursuant to a grant from the U.S. Social Security Administration (SSA) funded as part of the Retirement and Disability Research Consortium. The opinions and conclusions expressed are solely those of the author(s) and do not represent the opinions, or policy of SSA or any agency of the Federal Government. Neither the United States Government nor any agency thereof, nor any of their employees, makes any warranty, express or implied, or assumes any legal liability or responsibility for the accuracy, completeness, or usefulness of the contents of this report. Reference herein to any specific commercial product, process or service by trade name, trademark, manufacturer, or otherwise does not necessarily constitute or imply endorsement, recommendation or favoring by the United States Government or any agency thereof.

## Data Availability

The data that support the findings of this study are openly available in Panel Study of Income Dynamics at https://psidonline.isr.umich.edu/.

## Endnotes

^1^

https://www.ssa.gov/policy/docs/quickfacts/stat_snapshot/2023‐12.html (last accessed 08/29/2025). https://www.ssa.gov/policy/docs/statcomps/supplement/2024/highlights.html (last accessed 08/29/2025).

^2^

https://www.ssa.gov/policy/docs/quickfacts/stat_snapshot/2024‐12.html (last accessed 08/29/2025).

^3^

https://www.ssa.gov/oact/cola/sga.html (last accessed 08/29/2025). https://www.ssa.gov/benefits/disability/approval.html (last accessed 08/29/2025).

^4^

https://www.ssa.gov/disabilityresearch/wi/medicare.htm (last accessed 08/29/2025).

^5^

https://www.ssa.gov/oact/cola/sga.html (last accessed 08/29/2025).

^6^
Note that in this health production function, the health of an individual is conceptualized as a result of both systematic and random factors. The systematic component reflects the idea that work disability can often stem from accumulated poor health over time, capturing the non‐random accumulation or depletion of health capital leading to work disability. Conversely, the $\varepsilon_{t+1}$ term in the model represents random shocks to health, unforeseen events or conditions that can abruptly impact an individual's health status. Together, these elements underscore the dual nature of health outcomes: influenced by long‐term patterns and choices, yet also subject to sudden, unpredictable changes. Recognizing this dual nature is essential in understanding the complex dynamics of health and work disability.

^7^
See https://psidonline.isr.umich.edu/data/Documentation/UserGuide2023.pdf and https://www.bls.gov/cex/cecomparison/psid_profile.htm (last accessed on 08/30/2025).

^8^
These thresholds are based on the classification scheme in Meyer and Mok (2019) that builds on Charles (2003). In Meyer and Mok (2019), chronic disability is defined as three or more reports of any work disability during a 10‐year follow‐up. We adjust the threshold to 25% to better accommodate comparisons of individuals in their 50s prior to 1997 (i.e., with annual work disability reports) and those in their 50s after 1997 (i.e., with biennial reports). Mayer and Mok (2019) also use a 50% threshold to distinguish severe conditions versus non‐severe conditions.

^9^
In each wave, individuals are assigned a work disability score of zero (no limitations reported), one (mild limitations), two (moderate limitations), or three (severe limitations). Scores are aggregated across the observation period and divided by the maximum possible score based on the number of reports during the observation period (Jajtner 2020). For example, for individuals with 10 reports between ages 50 and 61, those with all moderate reports would have an index of 0.667, and those with six severe reports would have a score of 0.6. For individuals with five reports, just three reports would need to be severe to have an index value of 0.6.

^10^
We apply each state's statutory percent‐of‐federal multiplier to the federal maximum to form the state supplement. We do not adjust for whether the state credit is refundable vs. non‐refundable because our exposure measure is maximum statutory generosity (policy parameters), not realized/received benefits. Results are robust to excluding all state supplements (federal‐only exposure), indicating that inference does not hinge on the refundability distinction. See Section 5.4, Table 3 (“Federal EITC only”).

^11^
Thus, a co‐resident 19‐year‐old is not counted as a qualifying child for the parents' EITC (child count drops accordingly), while a 19‐year‐old who is a separate head/spouse has exposure based on their own household composition.

^12^
These category indicators (rather than a continuous count) ensure the empirical strategy follows a grouped DiD structure aligned with how federal EITC generosity is parameterized by family size.

^13^
DI reports (i.e., Medicare reports) in 2021 are included since there is a 2‐year delay in Medicare coverage following a DI award. Sensitivity analyses suggest that excluding 2021 data and 1970‐1971 birth cohorts does not meaningfully change our results.

^14^
Imprecision in the childhood‐exposure estimates is driven primarily by limited policy variation during childhood for these cohorts. In order to observe work disability at older ages, when it is more prevalent, individuals born after 1969 are excluded from the main results. By definition, no one in our sample has any EITC exposure before age 5, and only the youngest cohorts (1957–1969) are ever exposed in childhood. From 1975 through the late 1980s, real federal EITC generosity changed little; major expansions arrive only with the 1986 reform and especially the early‐1990s legislation, and most state supplements were either absent or small during our cohorts' childhood years. Consequently, cross‐cohort and within‐state variance in childhood exposure is low relative to adulthood, when the large federal expansions and widespread state adoptions generate substantial dispersion. Because each person contributes to both childhood and adulthood exposure, the estimation sample is the same across these specifications; the difference in precision reflects the variance of the exposure measure rather than the sample size. As visualized in Figure 1, average potential exposure is relatively flat before age 19 for these cohorts and rises sharply during the 1990s. We therefore interpret the childhood coefficients cautiously and note that additional policy variation or older follow‐up of later‐born cohorts will be needed to sharpen these estimates.

## Appendix A

**TABLE A1:** Effects of $1000 of EITC exposure on work disability.

	(1)	(2)	(3)	(4)
	Any limit ages 50–61	CS work limit ages 50–61	Work limit index ages 50–61	DI
EITC exposure (ages 0–49)	−0.00125**	−0.000943***	−0.000755***	−0.000835***
	(0.000423)	(0.000233)	(0.000195)	(0.000222)
Average age (ages 50–61)	0.585**	−0.0618	−0.151	
	(0.200)	(0.124)	(0.133)	
Average age2 (ages 50–61)	−0.00536**	0.000517	0.00132	
	(0.00188)	(0.00117)	(0.00125)	
Female (ref. Male)	0.0529***	−0.00297	0.00912	0.0118
	(0.0137)	(0.00799)	(0.00636)	(0.0119)
nH black (ref. nH white)	−0.0244	−0.0273	−0.0338*	0.0898**
	(0.0262)	(0.0245)	(0.0167)	(0.0289)
Hispanic (ref. nH white)	−0.0637*	−0.0153	−0.0472**	−0.0101
	(0.0281)	(0.0223)	(0.0133)	(0.0193)
Other r/*e* (ref. nH white)	−0.0541	0.00120	−0.0462	0.0522
	(0.0717)	(0.0500)	(0.0286)	(0.0420)
HS/GED (ref. < HS)	−0.0650	−0.0681**	−0.0367+	−0.0839+
	(0.0407)	(0.0230)	(0.0211)	(0.0433)
Some college (ref. < HS)	−0.0544	−0.0673*	−0.0254	−0.0802
	(0.0446)	(0.0254)	(0.0248)	(0.0563)
College (ref. < HS)	−0.114**	−0.102***	−0.0719**	−0.159**
	(0.0412)	(0.0225)	(0.0214)	(0.0490)
% Time married (ages 50–61)	−0.0223	−0.0509**	−0.0144	−0.00959
	(0.0230)	(0.0151)	(0.0141)	(0.0286)
% Time in poverty (ages 50–61)	0.323***	0.239***	0.254***	0.239***
	(0.0443)	(0.0374)	(0.0232)	(0.0508)
% Time married (ages 30–49)	−0.0173	0.00903	−0.0149	0.00405
	(0.0340)	(0.0206)	(0.0182)	(0.0338)
% Time in poverty (ages 0–49)	0.263***	0.163*	0.173***	0.290***
	(0.0720)	(0.0643)	(0.0482)	(0.0655)
Max EITC children = 1 (ref. = 0)	−0.00090	0.0235	0.0114	−0.00113
	(0.0354)	(0.0260)	(0.0202)	(0.0331)
Max EITC children = 2 (ref. = 0)	0.0220	0.0306	0.0130	0.0210
	(0.0373)	(0.0245)	(0.0179)	(0.0251)
Max EITC children = 3+ (ref. = 0)	0.0613+	0.0524+	0.0344*	0.0221
	(0.0334)	(0.0267)	(0.0169)	(0.0283)
Constant	−15.27**	2.228	4.618	0.482**
	(5.283)	(3.295)	(3.535)	(0.176)
Observations	5260	5260	5260	4449

*Note:* Corresponds to Figure 1. “Any Limit” is a binary dependent variable for any reported work limitation between ages 50 and 61. “CS Limit” is a binary characteristic for reported chronic and severe work limitations between ages 50 and 61. “Index” is the continuous work limitation index. “DI” is a binary variable for whether the individual likely had DI before age 65. All results are from an OLS regression model outlined in the methods section. All regressions include controls for state‐level environment characteristics between ages 0—49 (unemployment rate, minimum wage, income per capita, and hospital beds per capita), birth state fixed effects and birth cohort fixed effects, and the full control set described in Data and Method section, but non‐essential coefficient estimates are omitted from the displayed results for brevity. “Average age” refers to the average observed age when the outcome, work disability, is observed over ages 50—61. It is omitted for column 4, since DI receipt is observed as any report of Medicare coverage prior to age 65. “nH” refers to non‐Hispanic, “HS” refers to High School, “HS/GED” refers to High School or General Equivalency Diploma, “College” refers to reports of at least 4 or more years of college. + *p* < 0.1.

^*^ *p* < 0.05.

^**^ *p* < 0.01.

^***^ *p* < 0.001.

*Source:* Authors' calculations using PSID public data.

**TABLE A2:** Heterogeneous effects of $1000 of EITC exposure on work disability by race/ethnicity, sex, and educational attainment.

	(1)	(2)	(3)	(4)	(5)	(6)	(7)	(8)	(9)	(10)	(11)	(12)
	Ever work limit ages 50–59			CS work limit ages 50–59			Work limit index Ages50‐59			DI		
Interaction coefficients:												
Female x EITC (ref. Male)	0.00045			0.00023			0.00026			0.00048		
	(0.00037)			(0.00019)			(0.00021)			(0.00032)		
Black x EITC (ref. White)		−0.00121***			−0.00053+			−0.00073**			0.00016	
		(0.00033)			(0.00030)			(0.00024)			(0.00058)	
Hispanic x EITC (ref. White)		−0.00017			−0.00056+			−0.00034			−0.00051	
		(0.00053)			(0.00029)			(0.00025)			(0.00041)	
Other R/E x EITC (ref. White)		0.00006			0.00007			0.00023			0.00081	
		(0.00096)			(0.00068)			(0.00044)			(0.00056)	
HS x EITC (ref. < HS)			0.00175*			0.00116**			0.00101*			0.00042
			(0.00071)			(0.00036)			(0.00040)			(0.00083)
Some college x EITC (ref. < HS)			0.00108+			0.00088*			0.00074*			−0.00003
			(0.00060)			(0.00037)			(0.00030)			(0.00077)
College x EITC (ref. < HS)			0.00164**			0.00133**			0.00106***			0.00064
			(0.00052)			(0.00040)			(0.00027)			(0.00077)
Level coefficients:												
EITC ages 0–49	−0.00150**	−0.00108*	−0.00260***	−0.00108***	−0.00080***	−0.00196***	−0.00090***	−0.00062**	−0.00160***	−0.00110**	−0.00080**	−0.00118
	(0.00048)	(0.00048)	(0.00059)	(0.00025)	(0.00022)	(0.00047)	(0.00025)	(0.00020)	(0.00034)	(0.00031)	(0.00025)	(0.00082)
Female (ref. Male)	0.0292	0.0532***	0.0517***	−0.0153	−0.00329	−0.00447	−0.00439	0.00904	0.00805	−0.0145	0.0111	0.0113
	(0.0240)	(0.0137)	(0.0137)	(0.0148)	(0.00795)	(0.00807)	(0.0136)	(0.00636)	(0.00631)	(0.0220)	(0.0121)	(0.0118)
Black (ref. White)	−0.0241	0.0381	−0.0242	−0.0272	−0.000482	−0.0274	−0.0336+	0.00390	−0.0338+	0.0901**	0.0806	0.0897**
	(0.0263)	(0.0313)	(0.0262)	(0.0246)	(0.0344)	(0.0247)	(0.0166)	(0.0230)	(0.0168)	(0.0289)	(0.0478)	(0.0290)
Hispanic (ref. White)	−0.0634*	−0.0555	−0.0589*	−0.0151	0.0165	−0.0118	−0.0471**	−0.0283	−0.0443**	−0.00946	0.0206	−0.00907
	(0.0283)	(0.0455)	(0.0278)	(0.0224)	(0.0359)	(0.0225)	(0.0134)	(0.0226)	(0.0133)	(0.0193)	(0.0342)	(0.0196)
Other R/E (ref. White)	−0.0550	−0.0569	−0.0538	0.000698	−0.00303	0.000807	−0.0467	−0.0589	−0.0463	0.0513	0.00770	0.0523
	(0.0718)	(0.117)	(0.0705)	(0.0501)	(0.0823)	(0.0498)	(0.0287)	(0.0447)	(0.0281)	(0.0420)	(0.0594)	(0.0420)
HS (Ref. < HS)	−0.0657	−0.0629	−0.151**	−0.0684**	−0.0679**	−0.125***	−0.0371+	−0.0357+	−0.0862**	−0.0849+	−0.0848+	−0.107+
	(0.0404)	(0.0411)	(0.0490)	(0.0229)	(0.0224)	(0.0286)	(0.0210)	(0.0211)	(0.0242)	(0.0431)	(0.0438)	(0.0591)
Some college (ref. < HS)	−0.0561	−0.0517	−0.106+	−0.0683*	−0.0670*	−0.110**	−0.0264	−0.0242	−0.0609+	−0.0822	−0.0812	−0.0793
	(0.0440)	(0.0449)	(0.0577)	(0.0253)	(0.0248)	(0.0314)	(0.0246)	(0.0247)	(0.0303)	(0.0561)	(0.0566)	(0.0738)
College (ref. < HS)	−0.116**	−0.113**	−0.197***	−0.103***	−0.102***	−0.170***	−0.0729**	−0.0714**	−0.126***	−0.161**	−0.160**	−0.196**
	(0.0411)	(0.0415)	(0.0464)	(0.0224)	(0.0218)	(0.0291)	(0.0213)	(0.0212)	(0.0269)	(0.0487)	(0.0491)	(0.0646)
Observations	5260	5260	5260	5260	5260	5260	5260	5260	5260	4449	4449	4449

*Note:* Corresponds to Figure 2. “Any Limit” is a binary dependent variable for any reported work limitation between ages 50 and 61. “CS Limit” is a binary characteristic for reported chronic and severe work limitations between ages 50 and 61. “Index” is the continuous work limitation index. “DI” is a binary variable for whether the individual likely had DI before age 65. All results are from an OLS regression model outlined in the methods section. All regressions include controls detailed in Appendix Table A1 above, state‐level environment characteristics between ages 0—49 (unemployment rate, minimum wage, income per capita, and hospital beds per capita), birth state fixed effects and birth cohort fixed effects. “nH” refers to non‐Hispanic, “HS” refers to High School, “HS/GED” refers to High School or General Equivalency Diploma, “College” refers to reports of at least 4 or more years of college. + *p* < 0.1.

^*^ *p* < 0.05.

^**^ *p* < 0.01.

^***^ *p* < 0.001.

*Source:* Authors' calculations using PSID public data.

## References

[hec70068-bib-0001] Arrow, K. J. 1963. “Uncertainty and the Welfare of Economics of Medical Care.” American Economic Review 53, no. 5: 941–973.

[hec70068-bib-0002] Averett, S. , and Y. Wang . 2013. “The Effects of Earned Income Tax Credit Payment Expansion on Maternal Smoking.” Health Economics 22, no. 11: 1344–1359. 10.1002/hec.2886. 23239400

[hec70068-bib-0003] Bastian, J. 2020. “The Rise of Working Mothers and the 1975 Earned Income Tax Credit.” American Economic Journal: Economic Policy 12, no. 3: 44–75. 10.1257/pol.20180039.

[hec70068-bib-0004] Bastian, J. , and M. R. Jones . 2021. “Do EITC Expansions Pay for Themselves? Effects on Tax Revenue and Government Transfers.” Journal of Public Economics 196: 104355. 10.1016/j.jpubeco.2020.104355.

[hec70068-bib-0005] Bastian, J. , and L. Lochner . 2020. The EITC and Maternal Time Use: More Time Working and less Time with Kids?. National Bureau of Economic Research. https://www.nber.org/papers/w27717.

[hec70068-bib-0006] Bastian, J. , and K. Michelmore . 2018. “The Long‐Term Impact of the Earned Income Tax Credit on Children’s Education and Employment Outcomes.” Journal of Labor Economics 36, no. 4: 1127–1163. 10.1086/697477.

[hec70068-bib-0007] Boudreaux, M. H. , E. Golberstein , and D. D. McAlpine . 2016. “The Long‐Term Impacts of Medicaid Exposure in Early Childhood: Evidence From the Program’s Origin.” Journal of Health Economics 45: 161–175. 10.1016/j.jhealeco.2015.11.001. 26763123 PMC4785872

[hec70068-bib-0008] Braga, B. , F. Blavin , and A. Gangopadhyaya . 2020. “The long‐term Effects of Childhood Exposure to the Earned Income Tax Credit on Health Outcomes.” Journal of Public Economics 190: 104249. 10.1016/j.jpubeco.2020.104249.

[hec70068-bib-0009] Brown, C. 1996. “ *Notes on the ‘SEO’ or ‘Census’ Component of the PSID* (Technical Series Paper #96‐03).” Panel Study of Income Dynamics. https://psidonline.isr.umich.edu/publications/Papers/tsp/1996‐03_Notes_on_the_SEO_C_Brown.pdf.

[hec70068-bib-0010] Charles, K. K. 2003. “The Longitudinal Structure of Earnings Losses Among Work‐Limited Disabled Workers.” Journal of Human Resources 38, no. 3: 618–646. 10.3368/jhr.XXXVIII.3.618.

[hec70068-bib-0011] Chetty, R. , J. N. Friedman , and J. Rockoff . 2011. “New Evidence on the Long‐Term Impacts of Tax Credits.” Proceedings. Annual Conference on Taxation and Minutes of the Annual Meeting of the National Tax Association 104: 116–124.

[hec70068-bib-0012] Chetty, R. , J. N. Friedman , and E. Saez . 2013. “Using Differences in Knowledge Across Neighborhoods to Uncover the Impacts of the EITC on Earnings.” American Economic Review 103, no. 7: 2683–2721. 10.1257/aer.103.7.2683.

[hec70068-bib-0013] Cohen, S. , and D. Janicki‐Deverts . 2012. “Who’s Stressed? Distributions of Psychological Stress in the United States in Probability Samples from 1983, 2006, and 2009 ^1^ .” Journal of Applied Social Psychology 42, no. 6: 1320–1334. 10.1111/j.1559-1816.2012.00900.x.

[hec70068-bib-0014] Courtney‐Long, E. A. , D. D. Carroll , Q. C. Zhang , et al. 2015. “Prevalence of Disability and Disability Type Among Adults—United States, 2013.” MMWR. Morbidity and Mortality Weekly Report 64, no. 29: 777–782. 10.15585/mmwr.mm6429a2. 26225475 PMC4584831

[hec70068-bib-0015] Cowan, B. , and N. Tefft . 2012. “Education, Maternal Smoking, and the Earned Income Tax Credit.” B.E. Journal of Economic Analysis & Policy 12, no. 1. 10.1515/1935-1682.3305.

[hec70068-bib-0016] Dahl, G. B. , and L. Lochner . 2012. “The Impact of Family Income on Child Achievement: Evidence From the Earned Income Tax Credit.” American Economic Review 102, no. 5: 1927–1956. 10.1257/aer.102.5.1927.

[hec70068-bib-0017] Deshpande, M. , T. Gross , and Y. Su . 2019. Disability and Distress: The Effect of Disability Programs on Financial Outcomes. NBER Working Paper W25642.

[hec70068-bib-0018] Dickert, S. , S. Houser , and J. K. Scholz . 1995. “The Earned Income Tax Credit and Transfer Programs: A Study of Labor Market and Program Participation.” Tax Policy and the Economy 9: 1–50. 10.1086/tpe.9.20061826.

[hec70068-bib-0019] Dickert‐Conlin, S. , and S. Houser . 2002. “EITC and Marriage.” National Tax Journal 55, no. 1: 25–40. 10.17310/ntj.2002.1.02.

[hec70068-bib-0020] Dowd, T. , and J. B. Horowitz . 2011. “Income Mobility and the Earned Income Tax Credit: Short‐Term Safety Net or Long‐Term Income Support.” Public Finance Review 39, no. 5: 432–436. 10.1177/1091142111401008.

[hec70068-bib-0021] Eissa, N. , and H. Hoynes . 2006. “Behavioral Responses to Taxes: Lessons From the EITC and Labor Supply.” Tax Policy and the Economy 20: 73–110. 10.1086/tpe.20.20061905.

[hec70068-bib-0022] Eissa, N. , and J. B. Liebman . 1996. “Labor Supply Response to the Earned Income Tax Credit.” Quarterly Journal of Economics 111, no. 2: 605–637. 10.2307/2946689.

[hec70068-bib-0023] Erickson, P. 1998. “Evaluation of a Population‐Based Measure of Quality of Life: The Health and Activity Limitation Index (Halex).” Quality of Life Research 7, no. 2: 101–114. 10.1023/a:1008897107977. 9523491

[hec70068-bib-0024] Erickson, P. , R., Wilson , & I. I., Shannon 1995. Years of Healthy Life. 10.1037/e583992012-001 11762385

[hec70068-bib-0025] Ettner, S. L. 1996. “New Evidence on the Relationship Between Income and Health.” Journal of Health Economics 15, no. 1: 67–85. 10.1016/0167-6296(95)00032-1. 10157429

[hec70068-bib-0026] Evans, W. N. , and C. L. Garthwaite . 2014. “Giving Mom a Break: The Impact of Higher EITC Payments on Maternal Health.” American Economic Journal: Economic Policy 6, no. 2: 258–290. 10.1257/pol.6.2.258.

[hec70068-bib-0027] Gangopadhyaya, A. , F. Blavin , B. Braga , and J. Gates . 2020. “Credit Where It is Due: Investigating Pathways From Earned Income Tax Credit Expansion to Maternal Mental Health.” Health Economics 29, no. 9: 975–991. 10.1002/hec.4034. 32597518

[hec70068-bib-0028] Gardner, J. , and A. J. Oswald . 2007. “Money and Mental Wellbeing: A Longitudinal Study of Medium‐Sized Lottery Wins.” Journal of Health Economics 26, no. 1: 49–60. 10.1016/j.jhealeco.2006.08.004. 16949692

[hec70068-bib-0029] Golberstein, E. 2015. “The Effects of Income on Mental Health: Evidence From the Social Security Notch.” Journal of Mental Health Policy and Economics 18, no. 1: 27–37. PMID: 25862202, PMCID: PMC4494112. 25862202 PMC4494112

[hec70068-bib-0030] Goodman‐Bacon, A. 2021. “The Long‐Run Effects of Childhood Insurance Coverage: Medicaid Implementation, Adult Health, and Labor Market Outcomes.” American Economic Review 111, no. 8: 2550–2593. 10.1257/aer.20171671.

[hec70068-bib-0031] Grogger, J. 2003. “The Effects of Time Limits, the EITC, and Other Policy Changes on Welfare Use, Work, and Income Among Female‐Headed Families.” Review of Economics and Statistics 85, no. 2: 394–408. 10.1162/003465303765299891.

[hec70068-bib-0032] Grossman, M. 1972. “On the Concept of Health Capital and the Demand for Health.” Journal of Political Economy 80, no. 2: 223–255. 10.1086/259880.

[hec70068-bib-0033] Halliday, T. , B. Mazumder , and A. Wong . 2021. “Intergenerational Mobility in Self‐Reported Health Status in the US.” Journal of Public Economics 193: 104307. 10.1016/j.jpubeco.2020.104307. 33716349 PMC7948082

[hec70068-bib-0034] Hokayem, C. , and J. P. Ziliak . 2014. “Health, Human Capital, and Life Cycle Labor Supply.” American Economic Review 104, no. 5: 127–131. 10.1257/aer.104.5.127.

[hec70068-bib-0035] Hoynes, H. 2009. “The Earned Income Tax Credit, Welfare Reform, and the Employment of Low‐Skilled Single Mothers.” Strategies for Improving Economic Mobility of Workers: Bridging Theory and Practice MI: 65–78: *WE Upjohn Institute for Employment Research*. 10.17848/9781441631992.ch4.

[hec70068-bib-0036] Hoynes, H. , D. Miller , and D. Simon . 2015. “Income, the Earned Income Tax Credit, and Infant Health.” American Economic Journal: Economic Policy 7, no. 1: 172–211. 10.1257/pol.20120179.

[hec70068-bib-0037] Hoynes, H. , and A. J. Patel . 2018. “Effective Policy for Reducing Poverty and Inequality? The Earned Income Tax Credit and the Distribution of Income.” Journal of Human Resources 53, no. 4: 859–890. 10.3368/jhr.53.4.1115-7494R1.

[hec70068-bib-0038] Hoynes, H. , D. W. Schanzenbach , and D. Almond . 2016. “Long‐Run Impacts of Childhood Access to the Safety Net.” American Economic Review 106, no. 4: 903–934. 10.1257/aer.20130375.

[hec70068-bib-0039] IRS . 2023a. EITC Participation Rate by States Tax Years 2013 Through 2020. Internal Revenue Service. https://www.eitc.irs.gov/eitc‐central/participation‐rate‐by‐state/eitc‐participation‐rate‐by‐states.

[hec70068-bib-0040] IRS . 2023b. EITC Reports and Statistics. Internal Revenue Service. https://www.irs.gov/credits‐deductions/individuals/earned‐income‐tax‐credit/eitc‐reports‐and‐statistics#:~:text=Nationwide%20as%20of%20December%202022,about%20%2464%20billion%20in%20EITC.

[hec70068-bib-0041] Jahoda, M. 1981. “Work, Employment, and Unemployment: Values, Theories, and Approaches in Social Research.” American Psychologist 36, no. 2: 184–191. https://psycnet.apa.org/doi/10.1037/0003‐066X.36.2.184.

[hec70068-bib-0042] Jajtner, K. 2020. “Work‐Limiting Disability and Intergenerational Economic Mobility.” Social Science Quarterly 101, no. 5: 2001–2016. 10.1111/ssqu.12836. 33223571 PMC7676749

[hec70068-bib-0043] Jajtner, K. , D. L. Brucker , and S. Mitra . 2022. “Midlife Work Limitations are Associated With Lower Odds of Survival and Healthy Aging.” Journals of Gerontology: Serie Bibliographique 77, no. 4: 790–802. 10.1093/geronb/gbab214. PMC8974351 34791218

[hec70068-bib-0044] Jolly, N. A. 2013. “The Impact of work‐limiting Disabilities on Earnings and Income Mobility.” Applied Economics 45, no. 36: 5104–5118. 10.1080/00036846.2013.818212.

[hec70068-bib-0045] Jones, J. W. 2020. Essays on the Impacts of the Supplemental Nutrition Assistance Program.

[hec70068-bib-0046] Lenhart, O. 2019. “The Effects of Income on Health: New Evidence From the Earned Income Tax Credit.” Review of Economics of the Household 17, no. 2: 377–410. 10.1007/s11150-018-9429-x.

[hec70068-bib-0047] Lenhart, O. 2021. “Earned Income Tax Credit and Crime.” Contemporary Economic Policy 39, no. 3: 589–607. 10.1111/coep.12522.

[hec70068-bib-0048] Lupien, S. J. , B. S. McEwen , M. R. Gunnar , and C. Heim . 2009. “Effects of Stress Throughout the Lifespan on the Brain, Behaviour and Cognition.” Nature Reviews Neuroscience 10, no. 6: 434–445. 10.1038/nrn2639. 19401723

[hec70068-bib-0049] Maestas, N. , K. J. Mullen , and A. Strand . 2021. “The Effect of Economic Conditions on the Disability Insurance Program: Evidence From the Great Recession.” Journal of Public Economics 199: 104410. 10.1016/j.jpubeco.2021.104410. 34366496 PMC8340597

[hec70068-bib-0050] Markowitz, S. , K. A. Komro , M. D. Livingston , O. Lenhart , and A. C. Wagenaar . 2017. “Effects of State‐Level Earned Income Tax Credit Laws in the US on Maternal Health Behaviors and Infant Health Outcomes.” Social Science & Medicine 194: 67–75. 10.1016/j.socscimed.2017.10.016. 29073507 PMC5696026

[hec70068-bib-0051] Meyer, B. D. 2010. “The Effects of the Earned Income Tax Credit and Recent Reforms.” Tax Policy and the Economy 24, no. 1: 153–180. 10.1086/649831.

[hec70068-bib-0052] Meyer, B. D. , and W. K. C. Mok . 2019. “Disability, Earnings, Income and Consumption.” Journal of Public Economics 171: 51–69. 10.1016/j.jpubeco.2018.06.011.

[hec70068-bib-0053] Meyer, B. D. , and D. T. Rosenbaum . 2001. “Welfare, the Earned Income Tax Credit, and the Labor Supply of Single Mothers.” Quarterly Journal of Economics 116, no. 3: 1063–1114. 10.1162/00335530152466313.

[hec70068-bib-0054] Michelmore, K. , and L. M. Lopoo . 2021. “The Effect of Eitc Exposure in Childhood on Marriage and Early Childbearing.” Demography 58, no. 6: 2365–2394. 10.1215/00703370-9506903. 34568939

[hec70068-bib-0055] Miller, S. , and L. R. Wherry . 2019. “The long‐term Effects of Early Life Medicaid Coverage.” Journal of Human Resources 54, no. 3: 785–824. 10.3368/jhr.54.3.0816.8173R1.

[hec70068-bib-0056] Mitra, S. , D. L. Brucker , and K. Jajtner . 2020. “Wellbeing at Older Ages: Towards an Inclusive and Multidimensional Measure.” Disability and Health Journal 13, no. 4: 100926. 10.1016/j.dhjo.2020.100926. 32354618 PMC7541405

[hec70068-bib-0057] Moffitt, R. 1992. “Incentive Effects of the US Welfare System: A Review.” Journal of Economic Literature 30, no. 1: 1–61.

[hec70068-bib-0058] Muennig, P. A. , B. Mohit , J. Wu , H. Jia , and Z. Rosen . 2016. “Cost Effectiveness of the Earned Income Tax Credit as a Health Policy Investment.” American Journal of Preventive Medicine 51, no. 6: 874–881. 10.1016/j.amepre.2016.07.001. 27614902

[hec70068-bib-0059] NCSL . 2024. “Earned Income Tax Credit Overview.” National Conference of State Legislatures. https://www.ncsl.org/human‐services/earned‐income‐tax‐credit‐overview.

[hec70068-bib-0060] O’Connor, D. B. , J. F. Thayer , and K. Vedhara . 2021. “Stress and Health: A Review of Psychobiological Processes.” Annual Review of Psychology 72, no. 1: 663–688. 10.1146/annurev-psych-062520-122331. 32886587

[hec70068-bib-0061] PSID . 2022. Panel Study of Income Dynamics, Public Use Dataset. Produced and Distributed by the Survey Research Center. Institute for Social Research, University of Michigan.

[hec70068-bib-0062] Sareen, J. , T. O. Afifi , K. A. McMillan , and G. J. Asmundson . 2011. “Relationship Between Household Income and Mental Disorders: Findings From a Population‐Based Longitudinal Study.” Archives of General Psychiatry 68, no. 4: 419–427. 10.1001/archgenpsychiatry.2011.15. 21464366

[hec70068-bib-0063] Scholz, J. K. 1994. “The Earned Income Tax Credit: Participation, Compliance, and Antipoverty Effectiveness.” National Tax Journal 47, no. 1: 63–87. 10.1086/ntj41789053.

[hec70068-bib-0064] She, P. , and G. A. Livermore . 2007. “Material Hardship, Poverty, and Disability Among working‐age Adults.” Social Science Quarterly 88, no. 4: 970–989. 10.1111/j.1540-6237.2007.00513.x.

[hec70068-bib-0065] Shuey, K. M. , and A. E. Willson . 2017. “Trajectories of Work Disability and Economic Insecurity Approaching Retirement.” Journals of Gerontology: Serie Bibliographique 74, no. 7: 1200–1210. 10.1093/geronb/gbx096. PMC6748769 28977512

[hec70068-bib-0066] Siegel, J. J. 2021. Stocks for the Long Run: The Definitive Guide to Financial Market Returns & long‐term Investment Strategies. McGraw‐Hill Education.

[hec70068-bib-0067] Simon, D. , M. McInerney , and S. Goodell . 2018. “The Earned Income Tax Credit, Poverty and Health.” Health Affairs 10. 10.1377/hpb20180817.769687.

[hec70068-bib-0068] Social Security Administration . “ *Social Security Disability Facts*. Social Security Administration.” https://www.ssa.gov/disabilityfacts/facts.html.

[hec70068-bib-0069] Social Security Administration . 2019. *Annual Statistical Report on the Social Security Disability Insurance Program, 2018* (No. 13‐11826). Social Security Administration. https://www.ssa.gov/policy/docs/statcomps/di_asr/index.html.

[hec70068-bib-0070] Social Security Administration . 2022. *Annual Statistical Report on the Social Security Disability Insurance Program, 2021* (No. 13‐11826). Social Security Administration.

[hec70068-bib-0071] Social Security Administration . 2024. How you Qualify/Disability Benefits/SSA. Social Security Administration. https://www.ssa.gov/benefits/disability/qualify.html.

[hec70068-bib-0072] Social Security Administration (SSA) . 2018. “ *Disability Evaluation Under Social Security (Blue Book),* SSA Pub. No. 64‐039.” https://www.ssa.gov/disability/professionals/bluebook/.

[hec70068-bib-0073] Strully, K. W. , D. H. Rehkopf , and Z. Xuan . 2010. “Effects of Prenatal Poverty on Infant Health: State Earned Income Tax Credits and Birth Weight.” American Sociological Review 75, no. 4: 534–562. 10.1177/0003122410374086. 21643514 PMC3104729

[hec70068-bib-0074] University of Kentucky Center for Poverty Research . 2022. “UKCPR National Welfare Data, 1980‐2020.” http://ukcpr.org/resources/national‐welfare‐data.

[hec70068-bib-0075] Zechmann, A. , and K. I. Paul . 2019. “Why Do Individuals Suffer During Unemployment? Analyzing the Role of Deprived Psychological Needs in a Six‐Wave Longitudinal Study.” Journal of Occupational Health Psychology 24, no. 6: 641–661. 10.1037/ocp0000154. 30945924

